# SARS-CoV-2 Omicron BA.2.86 and JN.1 expand tropism in human proximal intestinal epithelium

**DOI:** 10.1038/s41467-026-74111-y

**Published:** 2026-06-05

**Authors:** Kenrie PY Hui, John CW Ho, Ka-Chun Ng, Jenny CM Chan, Taylor WC Ho, Rachel HH Ching, Leo LM Poon, Malik Peiris, John M Nicholls, Michael CW Chan

**Affiliations:** 1 School of Public Health, Li Ka Shing Faculty of Medicine, The University of Hong Kong, Pokfulam, Hong Kong China; 2 Centre for Immunology and Infection (C2I), Hong Kong Science Park, Hong Kong, China; 3 Department of Pathology, Li Ka Shing Faculty of Medicine, The University of Hong Kong, Hong Kong, China

**Keywords:** Infection, Viral pathogenesis, SARS-CoV-2

## Abstract

Omicron SARS-CoV-2 has diversified into multiple sub-lineages, complicating assessment of their intrinsic phenotypes due to background population immunity. We compare replication and biological characteristics of variants from BA.1 to JN.1 using human bronchial and lung explants, airway organoids, colon cells, and proximal intestinal enteroids. XBB.1.5 and EG.5.1 achieve higher replication titres in respiratory tissues than BA.2.86 and JN.1, indicating enhanced respiratory fitness. EG.5.1 displays dual cell-entry pathways and greater replication in alveolar epithelial cells, supporting increased lung tropism and pathogenicity. In contrast, BA.2.86 and JN.1 rely on TMPRSS2-mediated entry in airways. Notably, BA.2.86 and JN.1 replicate more efficiently than EG.5.1 in proximal intestinal enteroids in an ACE2- and TMPRSS2-dependent manner, but not in colon cells. JN.1 exhibits elevated intestinal tropism with limited proinflammatory cytokine induction, suggesting potential for faecal transmission. Here we show XBB.1.5 and EG.5.1 greater transmissibility and severity potential whereas BA.2.86 and JN.1 exhibit enhanced intestinal adaptation.

## Abstract (web summary)

EG.5.1 uses dual entry pathways and replicates better in alveolar cells. BA.2.86 and JN.1 depend on TMPRSS2 for airway entry but replicate more efficiently in intestinal enteroids, with JN.1 showing increased gut tropism and low inflammation.

## Introduction

Since the emergence of SARS-CoV-2 Omicron variants in November 2021, various descendant lineages, including BA.5, XBB (XBB.1.5 and EG.5.1), BA.2 (BA.2.86 and JN.1), have subsequently appeared and replaced the precursor strains. These mutant variants are characterized by enhanced viral fitness and immune evasion, hence posing huge challenge to impede the transmission of COVID-19^1^.

First reported in February 2023, EG.5 is a descendent lineage of XBB.1.9.2 with a spike protein F456L mutation. EG.5 and its sub-lineages, including EG.5.1, which carries an additional spike protein mutation Q52H, were designated as a variant of interest (VOI) in August 2023 by WHO, and have been circulated worldwide as the major strains as of November 2023^2^. EG.5.1 demonstrated an enhanced immune evasion from antibody neutralisation, which was attributed to its spike protein F456L mutation^3–5^. The global prevalence of EG.5 variants was gradually outcompeted by the emergence of BA.2.86 and its sub-lineages, including JN.1, which were subsequently classified as VOIs in November and December 2023^6^. BA.2.86 is a far descendant of the Omicron BA.2 lineage, and possesses 34 mutations in the spike protein relative to BA.2 and 36 mutations distinct from XBB.1.5^7^, whilst the JN.1 variant further carries a spike L455S mutation^8^. Nonetheless, both BA.2.86 and JN.1 variants displayed unprecedented immune evasive ability to the humoral immunity elicited by pre-XBB breakthrough infections, the monovalent and BA.1/BA.5 bivalent vaccinations^1,9–11^ leading to their high estimated reproduction numbers^11–13^.

Various types of COVID-19 vaccines, number of vaccinations and repeated infections generate the complex landscape of neutralising antibody levels and specificities of the community against Omicron variants making it difficult to evaluate the intrinsic phenotypes of the newly emerging SARS-CoV-2 variants solely based on clinical manifestations and epidemiological data.

**Fig. 1:**
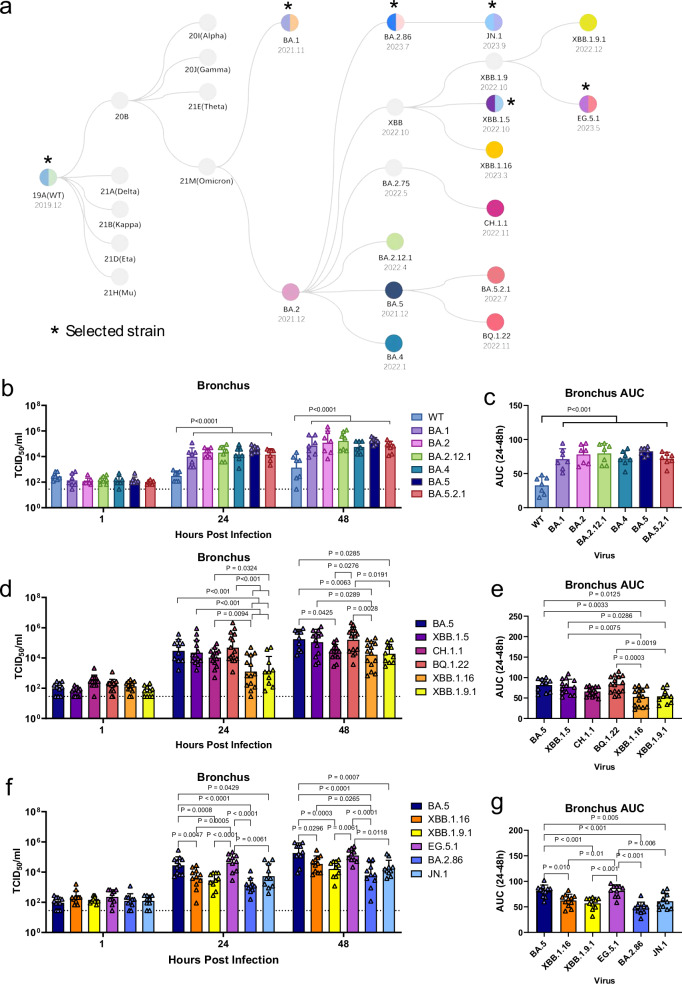
Phylogenetic relationship of SARS-CoV-2 variants and viral replication kinetics of SARS-CoV-2 variants in ex vivo cultures of human bronchial tissues. **a** A diagram showing the relationship of the SARS-CoV-2 variants. Virus strains not included in this study are in grey and virus strains tested in this study are in different colours. Dates are the first reported dates of the variant. * indicates selected strains for further investigation. **b**–**g** Replication of **b** wild-type (WT), BA.1, BA.2, BA.2.12.1, BA.4, BA.5, BA.5.2.1 (7 individual donors), **d** BA.5 (n = 10), BQ.1.22 (n = 15), CH.1.1 (n = 13), XBB.1.5 (n = 13), XBB.1.16 (n = 14) and XBB.1.9.1 (n = 10) (6 individual donors), **f** BA.5 (n = 11), XBB.1.16 (n = 11), XBB.1.9.1 (n = 10), EG.5.1 (n = 10), BA.2.86 (n = 10) and JN.1 (n = 10) (6 individual donors) in ex vivo cultures of human bronchi. Viral titres in culture supernatants are shown at the indicated times. **c**, **e** and **g** Viral titres from **b**, **d** and **f** are depicted as area under the curve (AUC). Bar-charts show the mean value ± standard deviation (SD). The horizontal dotted line denotes the limit of detection in the TCID_50_ assay. **b**, **d** and **f**, Statistical significance was calculated using two-way ANOVA followed by Tukey’s multiple comparison test; **c**, **e** and **g**, Statistical significance was calculated using one-way ANOVA followed by Tukey’s multiple comparison test. *P* < 0.05 was considered to be statistically significant, and exact *P* values are presented.

Here, we used physiologically relevant ex vivo explant cultures of human respiratory tissues, human airway organoids, proximal intestinal enteroids, and colon cells to evaluate the tissue tropism and viral replication of a number of dominant strains emerging between December 2021 and April 2024. The phylogenetic relationship of the viruses is illustrated in Fig. 1a. The dependence of host factors for the replication of EG.5.1, BA.2.86 and JN.1 in human intestine were studied in proximal intestinal enteroids and colon cells. We show that XBB.1.5 and EG.5.1 replicate to higher titres than BA.2.86 and JN.1 in human bronchial and lung explants, indicating superior respiratory fitness. In airway models, replication of BA.2.86 and JN.1, but not EG.5.1, is inhibited by TMPRSS2 blockade, suggesting differential entry mechanisms. Conversely, BA.2.86 and JN.1 replicate more efficiently in proximal intestinal enteroids than EG.5.1 does, in an ACE2-dependent manner, a pattern not observed in colon cells. Among the variants tested, BA.5, BA.5.2.1, XBB.1.5, and EG.5.1 exhibit intrinsically greater potential for transmission and disease severity. EG.5.1 demonstrates enhanced lung tropism and pathogenicity, likely due to dual cell-entry pathways and efficient replication in alveolar type I and II cells. In proximal intestinal enteroids, replication of BA.2.86 and JN.1 requires both ACE2 and TMPRSS2. Notably, JN.1 displays increased intestinal tropism with limited proinflammatory cytokine induction, which may facilitate viral dissemination via the faecal route.

## Results

### Comparison of viral replication in respiratory explants

According to the emergence of Omicron variants, we first compared the virus replication of a number of Omicron BA variants (including BA.1, BA.2, BA.2.12.1, BA.4, BA.5 and BA.5.2.1) with the wild-type (WT) strain as one batch, BA.5 together with several XBB variants, CH.1.1 and BQ.1.22 in another batch. Finally, we investigated the virus replication of EG.5.1, BA.2.86 and JN.1 in comparison with BA.5, XBB.1.16 and XBB.1.9.1 in the human ex-vivo cultures of bronchial tissues by quantifying infectious virus titres.

All BA variants replicated significantly to higher titres than WT at both 24 hpi and 48 hpi (Fig. 1b) and area-under-curve (AUC) values of all tested BA variants were significantly higher than that of WT (Fig. 1c). When comparing the XBB variants with BA.5, CH.1.1, and BQ.1.22, XBB.1.16 and XBB.1.9.1 had significantly lower titres than the other 4 variants (BA.5, XBB.1.5, CH.1.1 and BQ.1.22) at 24 hpi, while BA.5, XBB.1.5 and BQ.1.22 had relatively higher titres than CH.1.1, XBB.1.16 and XBB.1.9.1 at 48 hpi (Fig. 1d). The AUC levels of BA.5, XBB.1.5 and BQ.1.22 show similar trends as the replication of 48 hpi, which were higher than that of CH.1.1, XBB.1.16 and XBB.1.9.1 (Fig. 1e). Direct comparison of EG.5.1, BA.2.86 and JN.1 demonstrates a similar replication competence of EG.5.1 to BA.5, which was significantly higher than that of BA.2.86, JN.1, XBB.1.16 and XBB.1.9.1 (Fig. 1f, g). Taken together the three batches of comparison, BA variants (BA.1, BA.2, BA.4, BA.5 and BA.5.2.1), XBB.1.5, BQ.1.22 and EG.5.1 demonstrated higher tropism in human bronchial tissues than the other tested variants including the currently dominated strains of BA.2.86 and JN.1, suggesting that these variants are able to transmit between humans more efficiently than the others.

**Fig. 2:**
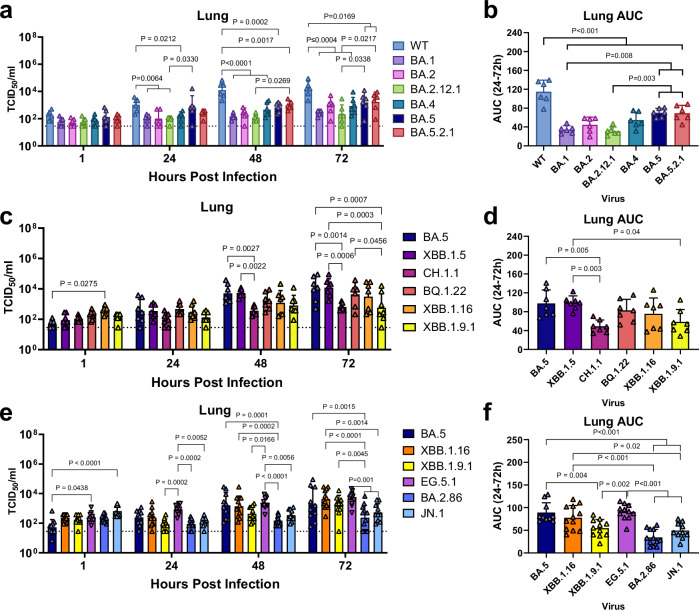
Viral replication kinetics of SARS-CoV-2 variants in ex vivo cultures of human lung explants. Replication of **a** wild-type (WT), BA.1, BA.2, BA.2.12.1, BA.4, BA.5, BA.5.2.1 (6 individual donors), **c** BA.5, BQ.1.22, CH.1.1, XBB.1.5, XBB.1.16 and XBB.1.9.1 (7 individual donors), **e** BA.5 (n = 10), XBB.1.16 (n = 12), XBB.1.9.1 (n = 11), EG.5.1 (n = 11), BA.2.86 (n = 12) and JN.1 (n = 10) (6 individual donors) in ex vivo cultures of human lung explants. Viral titres in culture supernatants are shown at the indicated times.** b**,** d** and **f** Viral titres from **a**, **c** and **e** are depicted as area under the curve (AUC). Bar-charts show the mean value ± standard deviation (SD). The horizontal dotted line denotes the limit of detection in the TCID_50_ assay. **a**, **c** and **e**, Statistical significance was calculated using two-way ANOVA followed by Tukey’s multiple comparison test; **b**, **d** and **f**, Statistical significance was calculated using one-way ANOVA followed by Tukey’s multiple comparison test. *P* < 0.05 was considered to be statistically significant, and exact *P* values are presented.

In lung explants, WT replicated to the highest titres at 24 hpi, 48 hpi and 72 hpi than all the tested Omicron BA subvariants (Fig. 2a). We also observed that BA.2.12.1 replicated to lower titres than BA.5 at 24 and 72 hpi while lower than BA.5.2.1 at 48 and 72 hpi (Fig. 2a). The highest replication competence of WT was confirmed by the AUC level and both BA.5 and BA.5.2.1 had a significantly higher AUC levels than BA.1 and BA.2.12.1 (Fig. 2b). When comparing with XBB subvariants, BA.5 and XBB.1.5 replicated to higher titres than CH.1.1 at 48 and 72 hpi (Fig. 2c) while BA.5, XBB.1.5 and BQ.1.22 replicated to higher titres than XBB.1.9.1 at 72 hpi (Fig. 2c). AUC levels of BA.5 and XBB.1.5 confirmed the higher replication competence than CH.1.1, while XBB.1.5 had a higher AUC level than XBB.1.9.1 (Fig. 2d).

EG.5.1 consistently replicated to higher titres than BA.2.86 and JN.1 from 24 to 72 hpi (Fig. 2e). BA.2.86 replicated to lower titres than BA.5, XBB.1.16 and XBB.1.9.1 at 48 and 72 hpi, while JN.1 had a lower titre than XBB.1.16 (Fig. 2e). Higher replication competence of EG.5.1, BA.5 and XBB.1.16 than BA.2.86 and JN.1 was confirmed by the AUC levels (Fig. 2f). Besides, EG.5.1 and BA.5 had a higher AUC level than XBB.1.9.1 (Fig. 2f). Taken together, the findings on lung explants, WT has the highest replication competence in human lungs when compared to the Omicron subvariants. More importantly, among all the Omicron subvariants tested, BA.5, XBB.1.5, EG.5.1 and XBB.1.16 replicated to higher titres than BA.1, BA.2.12.1, CH.1.1, XBB.1.9.1, BA.2.86 and JN.1 while BQ.1.22 had the medium replication competence.

### Cellular tropism in human bronchial and lung explants

**Fig. 3:**
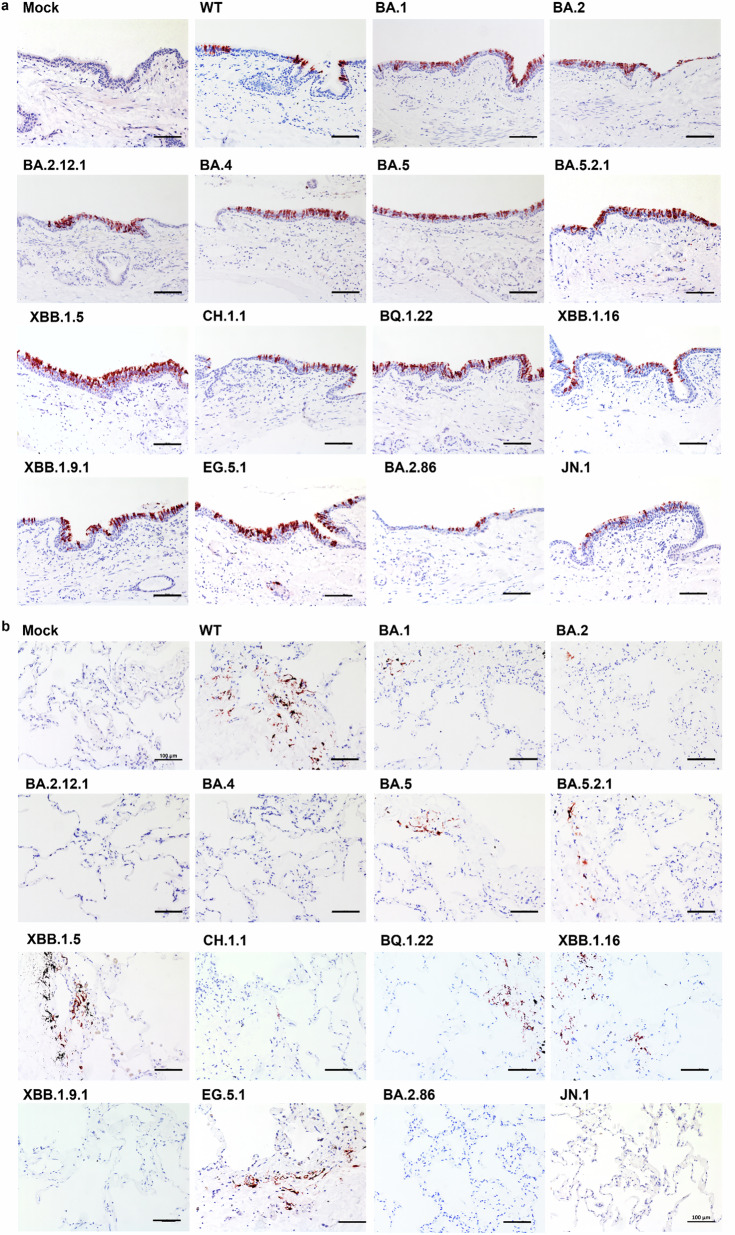
Tissue tropism of SARS-CoV-2 variants in ex vivo cultures of human respiratory tissues. Distribution of virus-infected cells of all tested variants in ex vivo cultures of human **a** bronchus and **b** lung explants. The tissues were formalin-fixed at 48 or 72 hpi for **a** bronchi and **b** lung, respectively. Immunohistochemical staining of SARS-CoV-2 nucleocapsid protein is identified as a red-brown colour. The images are representatives of two individual donors. Scale bars, 100 μm.

Immunohistochemical staining of viral nucleocapsid (N) protein showing the replication competence of the subvariants in the human bronchial and lung explants (Fig. 3a, b). More extensive staining of N protein was observed in BA.1, BA.2, BA.4, BA.5, BA.5.2.1, XBB.1.5, BQ.1.22 and EG.5.1 than WT, BA.2.86 and JN.1 in human bronchial tissues while more extensive staining were observed in WT, XBB.1.5, EG.5.1 and XBB.1.16 infection than the other variants in human lung explants (Fig. 3b). The extensiveness of viral protein staining was in concordance with the replication competence of the subvariants.

**Fig. 4:**
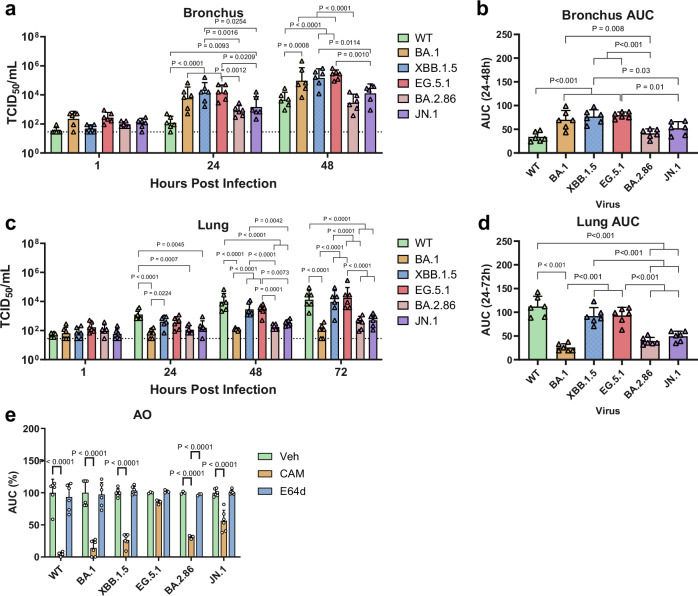
Viral replication kinetics of SARS-CoV-2 variants in ex vivo cultures of human bronchial and lung explants and dependence on TMPRSS2 and cathepsins of host cell. Replication of wild-type (WT), BA.1, XBB.1.5, EG.5.1, BA.2.86 and JN.1 was directly compared in ex vivo cultures of human **a** bronchi and **c** lung explants (6 individual donors). Virus titres in culture supernatants are shown. **b**, **d** Viral titres from **a** and **c** are depicted as the area under the curve (AUC). Bar-charts show the mean value ± standard deviation (SD). The horizontal dotted line denotes the limit of detection in the TCID_50_ assay. **e** Replication of WT, BA.1, XBB.1.5, EG.5.1, BA.2.86 and JN.1 in human airway organoids (3 individual donors) in the presence of camostat mesylate (CAM), E64d or vehicle (Veh). Replication is depicted as a percentage of AUC with reference to the AUC of the corresponding Veh in the bar-chart showing the mean value ± standard deviation (SD). **a**, **c **Statistical significance was calculated using two-way ANOVA followed by Tukey’s multiple comparisons test; **b**, **d** and **e**, Statistical significance was calculated using one-way ANOVA followed by Tukey’s multiple comparisons test. *P* < 0.05 was considered to be statistically significant, and exact *P* values are presented.

From the panel of SARS-CoV-2 viruses, six representative strains (WT, BA.1, XBB.1.5, EG.5.1, BA.2.86 and JN.1) from different periods were selected based on their clinical significance for further in-depth analysis. BA.1, XBB.1.5 and EG.5.1 had replication competences higher than WT and BA.2.86 while XBB.1.5 and EG.5.1 replicated more efficiently than JN.1 in bronchial explants (Fig. 4a, b). In lung explants, WT, XBB.1.5 and EG.5.1 had a higher replication competence than BA.1, BA.2.86 and JN.1 (Fig. 4c, d).

**Fig. 5:**
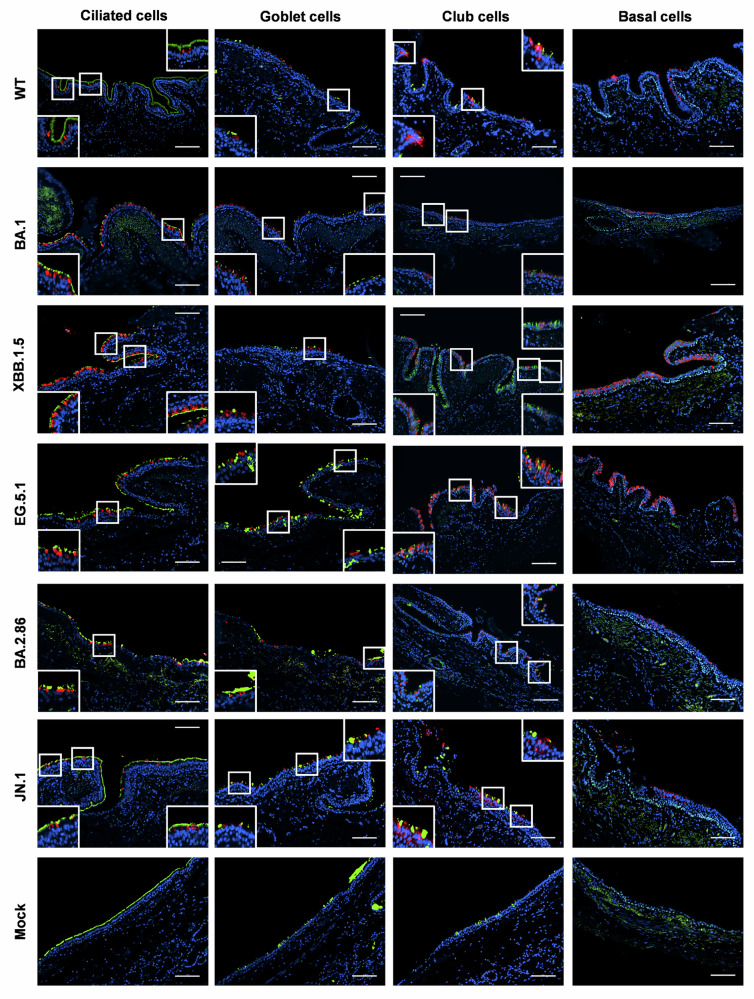
Cellular tropism of SARS-CoV-2 variants in ex vivo cultures of human bronchus. Cellular tropism of wild-type (WT), BA.1, XBB.1.5, EG.5.1, BA.2.86 and JN.1 in ex vivo cultures of the respiratory tract. Infected bronchi were fixed at 48 hpi and stained with SARS-CoV-2 viral protein in red and indicated cell markers in green: acetyl-α-tubulin for ciliated cells, mucin 5AC for secretory goblet cells, club cell protein 10 for club cells and p63-α for basal cells; 4′,6-diamidino-2-phenylindole-positive for nuclei in blue. The images are representative of two individual donors. Scale bar, 100 μm.

**Fig. 6:**
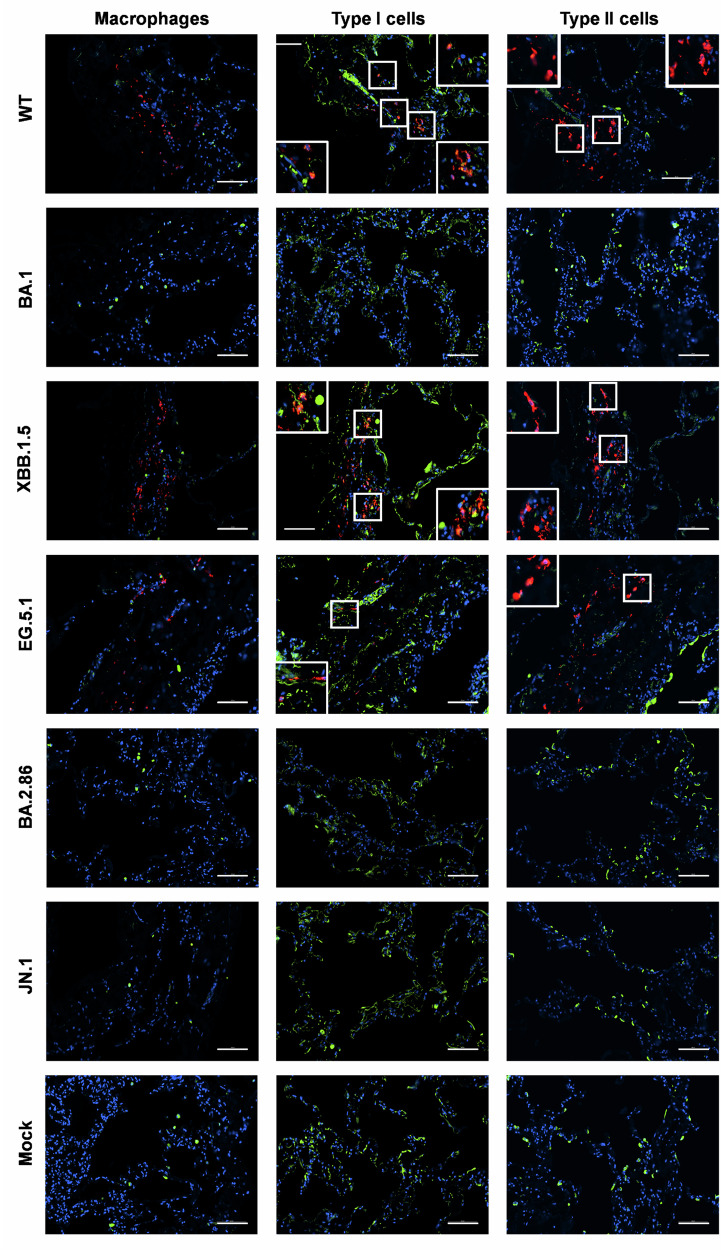
Cellular tropism of SARS-CoV-2 variants in ex vivo cultures of human lung explants. Cellular tropism of wild-type (WT), BA.1, XBB.1.5, EG.5.1, BA.2.86 and JN.1 in ex vivo cultures of the respiratory tract. Infected lung explants were fixed at 72 hpi and stained with SARS-CoV-2 viral protein in red and indicated cell markers in green: CD68 for macrophages, AGER for type I cells and HT2-280 for type II cells; 4′,6-diamidino-2-phenylindole-positive for nuclei in blue. The images are representative of two individual donors. Scale bar, 100 μm.

All six representative viruses showed similar cellular tropism in human bronchial epithelium with colocalization of viral N protein staining with cell markers of ciliated, goblet and club cells, while there was no co-staining observed in basal cells (Fig. 5). Co-staining of viral N protein with type I and type II cell markers was observed in WT, XBB.1.5 and EG.5.1 infected lung explants (Fig. 6), while no co-staining was found in macrophages. As limited infection by BA.1, BA.2.86 or JN.1, there were no viral N protein staining found in lung explants of these viruses.

### Differential dependence of TMPRSS2 in airway organoids

In order to investigate the preference of entry route of SARS-CoV-2 viruses, we applied camostat mesylate (CAM) and E64d to inactivate TMPRSS2 and cathepsins, respectively, in both Vero E6-T2 cells and human airway organoids (AO). In Vero E6-T2 cells, BA.2.86 replicated to lower titres in CAM-treated cells than vehicle- or E64d-treated cells at 24 hpi (Supplementary Fig. 1a), but there were no significant differences of AUC values of BA.2.86 and EG.5.1 in the presence of CAM or E64d (Supplementary Fig. 1b). In contrast, significant reduction of viral titres of BA.2.86 was observed in AO treated with CAM when compared to titres in AO treated with vehicle of E64d at both 24 and 48 hpi (Supplementary Fig. 1c). The AUC levels of BA.2.86 dramatically reduced by the treatment of CAM while titres of EG.5.1 were reduced by CAM treatment at 24 hpi compared to vehicle and E64d treatment and a lesser extent of reduction of AUC levels was observed in AO infected with EG.5.1 in the presence of CAM (Supplementary Fig. 1d). We found that comparable ACE2 expression levels in AO and bronchus while higher TMPRSS2 expression was detected in AO (Supplementary Fig. 2a–c). Therefore, we further investigated the preference of entry route of the representative viruses in AO. The replication of all tested viruses was not affected by E64d treatment (Fig. 4e). Various levels of replication reduction were observed by CAM treatment. The replication of WT and BA.1 was reduced to 5% and 14% in the presence of CAM, respectively, while XBB.1.5, BA.2.86 and JN.1 replication were reduced to 27%, 31% and 57% by CAM, respectively. EG.5.1 was the least sensitive to CAM treatment in AO.

### Replication competence in proximal intestinal enteroids

The replication competence and immune responses of the six representative viruses were compared in both human colorectal cancer Caco-2 cells and tissue-derived intestinal enteroids. Through direct comparison of the cellular markers, the intestinal enteroids were characterized with elevated expression of proximal intestinal cell markers, including lysozyme (LYZ), villin 1 (VIL1) and GATA binding protein 4 (GATA4), while Caco-2 cells had upregulated GATA binding protein 6 (GATA 6), which is expressed in colorectal epithelium (Supplementary Fig. 3a and b). Therefore, intestinal enteroids and Caco-2 cells represent proximal small intestine (proximal intestinal enteroids) and distal large intestine (colon cells), respectively. We detected higher mRNA expression of *ACE2-L* in the proximal intestinal enteroids than colon cells and comparable levels of *TMPRSS2* in the two models (Supplementary Fig. 2a). The protein expression of ACE2-L and TMPRSS2 was visualised by immunofluorescence staining (Supplementary Fig. 2b, c).

**Fig. 7:**
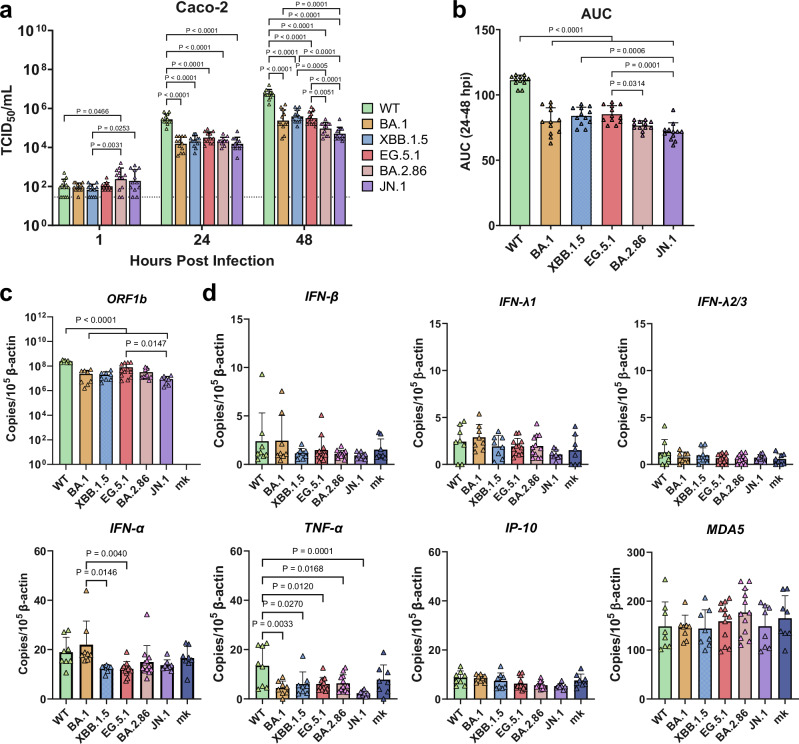
Viral replication kinetics of SARS-CoV-2 variants and the induction of innate immune responses in colon cells. **a** Replication of wild-type (WT), BA.1, XBB.1.5, EG.5.1, BA.2.86 and JN.1 in Caco-2 (colon) cells. Virus titres in culture supernatants are shown (n = 12). **b** Viral titres from **a** are depicted as the area under the curve (AUC). Bar-charts show the mean value ± standard deviation (SD) (n = 12). The horizontal dotted line denotes the limit of detection in the TCID_50_ assay. **c** mRNA expression of viral SARS-CoV-2 *ORF1b* gene and **d** innate immune responses (*IFN-β, IFN-λ1, IFN-λ2/3, IFN-α, TNF-α, IP-10 and MDA5*) of wild-type (WT) (n = 8), BA.1 (n = 8), XBB.1.5 (n = 8), EG.5.1 (n = 12), BA.2.86 (n = 12), JN.1 (n = 8) and mock infection (n = 8) were measured at 48 hpi. The mRNA copies expressed were normalised with 1 × 10^5^ β-actin copies. Data are the mean ± SD. **a** Statistical significance was calculated using two-way ANOVA followed by Tukey’s multiple comparisons test. **b**–**d** Statistical significance was calculated using one-way ANOVA followed by Tukey’s multiple comparisons test. *P* < 0.05 was considered to be statistically significant, and exact *P* values are presented.

WT replicated to the highest titres among the six viruses at 24 and 48 hpi (Fig. 7a) and highest AUC value (Fig. 7b). XBB.1.5 and EG.5.1 replicated to higher titres than BA.2.86 and JN.1 at 48 hpi (Fig. 7a). The AUC level of EG.5.1 was significantly higher than that of BA.2.86 and JN.1 while XBB.1.5 had a higher AUC value than JN.1 (Fig. 7b). Besides, WT had the highest level of viral ORF1b gene expression (Fig. 7c). WT induced higher levels of *TNF-α* in colon cells than all the other tested viruses while BA.1 induced higher *IFN-α* expression than XBB.1.5 and EG.5.1 (Fig. 7d).

**Fig. 8:**
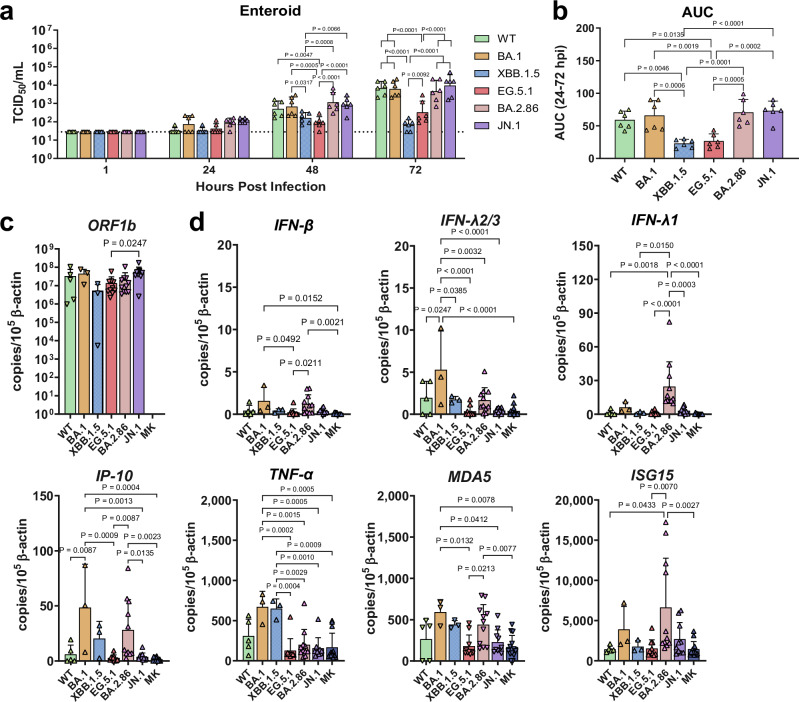
Viral replication kinetics of SARS-CoV-2 variants and the induction of innate immune responses in proximal intestinal enteroids. **a** Replication of wild-type (WT), BA.1, XBB.1.5, EG.5.1, BA.2.86 and JN.1 in proximal intestinal enteroids. Virus titres in culture supernatants are shown (n = 6) (3 individual donors). **b** Viral titres from **a** are depicted as area under the curve (AUC). Bar-charts show the mean value ± standard deviation (SD) (n = 6). The horizontal dotted line denotes the limit of detection in the TCID_50_ assay. **c** mRNA expression of viral SARS-CoV-2 *ORF1b* gene and **d** innate immune responses (*IFN-β, IFN-λ2/3, IFN-λ1, IP-10, TNF-α, MDA5 and ISG15*) of wild-type (WT) (n = 5), BA.1 (n = 3), XBB.1.5 (n = 3), EG.5.1 (n = 10), BA.2.86 (n = 11), JN.1 (n = 10) and mock infection (n = 13) (3 individual donors) were measured at 72 hpi. The mRNA copies expressed were normalised with 1 × 10^5^ β-actin copies. Data are the mean ± SD. **a** Statistical significance was calculated using two-way ANOVA followed by Tukey’s multiple comparisons test. **b**–**d** Statistical significance was calculated using one-way ANOVA followed by Tukey’s multiple comparisons test. *P* < 0.05 was considered to be statistically significant, and exact *P* values are presented.

In proximal intestinal enteroids, WT, BA.1, BA.2.86 and JN.1 replicated to higher titres than XBB.1.5 and EG.5.1 at 48 and 72 hpi, except that no statistical significance was measured between WT and XBB.1.5 at 48 hpi (Fig. 8a). These trends were confirmed by the values of AUC (Fig. 8b). JN.1 infection led to higher mRNA expression of viral *ORF1b* gene than EG.5.1 infection (Fig. 8c). BA.1 infection induced higher expression of *IFN-β, IP-10* and *MDA5* than EG.5.1 infection, higher levels of *MDA5* than JN.1 infection and the highest levels of *IFN-λ2/3* among the tested viruses (Fig. 8d). Moreover, BA.1 and XBB.1.5 induced higher expressions of *TNF-α* than EG.5.1, BA.2.86 and JN.1. BA.2.86 up-regulated more *IFN-β, ISG15* and *MDA5* than EG.5.1, induced the highest levels of *IFN-λ1* among the tested viruses except BA.1 and BA.2.86 also induced higher expression of *ISG15* than WT.

**Fig. 9:**
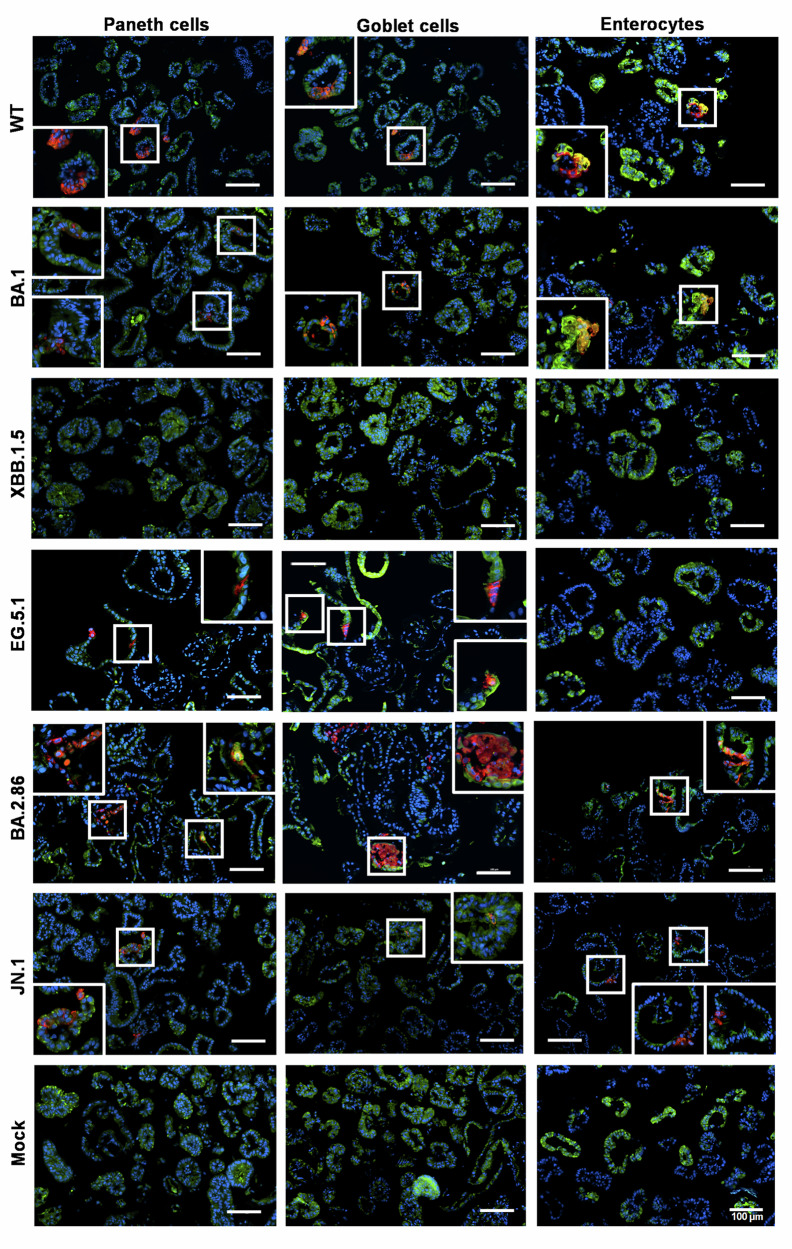
Cellular tropism of SARS-CoV-2 variants in proximal intestinal enteroids. Cellular tropism of wild-type (WT), BA.1, XBB.1.5, EG.5.1, BA.2.86 and JN.1 in proximal intestinal enteroids. Infected enteroids were fixed at 72 hpi and stained with SARS-CoV-2 viral protein in red and indicated cell markers in green: lysozyme for Paneth cells, mucin-2^+^ for goblet cells and FABP1 for enterocytes; 4′,6-diamidino-2-phenylindole-positive for nuclei in blue. The images are representatives of two individual donors. Scale bars, 100 μm.

Cellular tropism of WT and Omicron variants in the human proximal intestinal enteroids were visualised through immunofluorescence staining of the SARS-CoV-2 viral protein and cell markers of proximal intestinal enteroids, including cytosol lysozyme (LYZ) for Paneth cells, mucin-2 (MUC2) for goblet cells, and fatty acid binding protein (FABP1) for enterocytes (Fig. 9). Co-localisation of SARS-CoV-2 N protein foci were observed in the Paneth cells, goblet cells and enterocytes under infections of WT, BA.1, EG.5.1, BA.2.86, and JN.1, except for the absence of viral N protein staining in XBB.1.5-infected cells.

### Dependence of ACE2 and TMPRSS2 in enteroids and colon cells

**Fig. 10:**
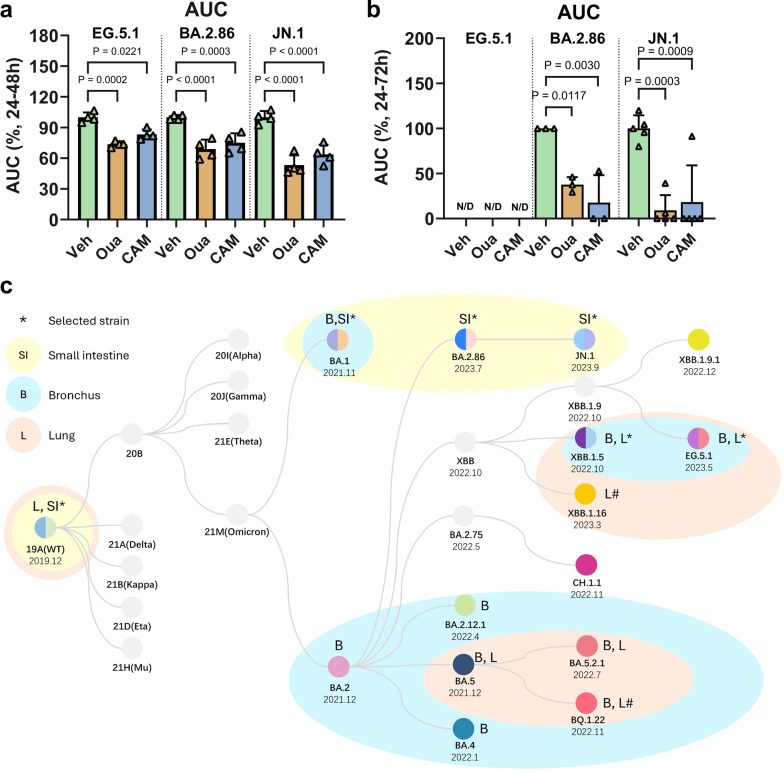
Dependence on ACE2 and TMPRSS2 of EG.5.1, BA.2.86 and JN.1 for infection in colon cells and proximal intestinal enteroids and summary of tissue tropism of SARS-CoV-2 variants. **a** Replication of EG.5.1 (n = 4), BA.2.86 (n = 4) and JN.1 (n = 4) in colon cells in the presence of ouabain (Oua), camostat mesylate (CAM) or vehicle (Veh). **b** Replication of EG.5.1 (n = 3), BA.2.86 (n = 3) and JN.1 (n = 5) in proximal intestinal enteroids (3 individual donors) in the presence of ouabain (Oua), camostat mesylate (CAM) or vehicle (Veh). Replication is depicted as a percentage of the AUC relative to the AUC of the corresponding Veh, with the bar chart showing the mean ± standard deviation (SD). N/D: non-detectable. **a**, **b** Statistical significance was calculated using one-way ANOVA followed by Tukey’s multiple comparisons test. *P* < 0.05 was considered to be statistically significant, and exact *P* values are presented. **c** Virus strains with relatively high replication competence in ex vivo bronchus are denoted with a blue oval, in lung explants are denoted with an orange oval and in proximal intestinal enteroids are denoted with ovals in yellow. * Selected strains for direct comparison of replication competence in all three above-mentioned models. # Strain with a trend of high replication competence in lung explants.

TMPRSS2 inhibitor (CAM) and ACE2 inhibitor (Oua) were applied in both colon cells and proximal intestinal enteroids infected with EG.5.1, BA.2.86 and JN.1. In colon cells, JN.1 was the most sensitive to both inhibitors compared to EG.5.1 and BA.2.86 while reduction percentages of BA.2.86 replication was slightly higher than EG.5.1 in the presence of the inhibitors (Fig. 10a). In proximal intestinal enteroids, with higher ACE2 receptor expression than colon cells (Supplementary Fig. 2a), TMPRSS2 inhibitor had similar levels of inhibition in viral replication of BA.2.86 and JN.1 while ACE2 inhibitor reduced the replication of JN.1 to 9.06% ± 17.02% compared to 37.69% ± 8.37% of BA.2.86 (Fig. 10b). EG.5.1 had limited replication in proximal intestinal enteroids with undetectable replication. These findings suggest that replication of JN.1 and BA.2.86 is more ACE2 and TMPRSS2 dependent in proximal intestinal enteroids than in colon cells and ACE2 binding is more essential to JN.1 replication in proximal intestinal enteroids than BA.2.86.

## Discussion

Vaccination and XBB breakthrough infection sera have lower neutralisation titres against BA.2.86 than EG.5.1^11,12^, while JN.1 has an even higher immune evasion ability than BA.2.86^10,13^. The immune evasion properties may mask the intrinsic virological characteristics of SARS-CoV-2 variants, including EG.5.1, BA2.86 and JN.1, during human infection.

By using ex vivo human respiratory explants, we found that BA.5, XBB.1.5, EG.5.1 and BA.5.2.1 had relatively high replication competence in both bronchial and lung explants, BA.1, BA.2, BA.2.12.1, BA.4 and BQ.1.22 replicating more efficiently in the bronchial explants, while WT and XBB.1.16 replicated better in lung explants. The tissue tropism of these Omicron variants has been summarised in Fig. 10c. Cellular tropism of WT, BA.1, XBB.1.5, EG.5.1, BA.2.86 and JN.1 was similar among these viruses with viral antigen stained with ciliated, goblet and club cells but not in basal cells in bronchial epithelium. WT, XBB.1.5 and EG.5.1 infected both alveolar type I and II cells but not macrophages in lung explants. We also show that the replication competence of EG.5.1 was much higher than that of BA.2.86 and JN.1 in human bronchus and lung explants. This may be attributed to the ability of EG.5.1 but not BA.2.86 or JN.1 to enter cells via both plasma membrane and endocytosis pathways, which were observed in our airway organoid (AO) experiments. JN.1 and BA.2.86, but not EG.5.1, are more TMPRSS2-dependent for cell entry in airway epithelium which is consistent with others reports on TMPRSS2-dependent pathway of BA.2.86 in lung cells^7,14^. The use of dual-entry pathways, the elevated lung tropism of EG.5.1, and infection in both alveolar type I and II cells suggest that EG.5.1 probably causes more severe lung disease than BA.2.86 and JN.1 infections. This implication is supported by a hamster study showing the lower pathogenicity of BA.2.86 and lower ability to breach the barriers between airway epithelium and endothelium than EG.5.1^12^. The discrepancies in the results performed in Vero E6-T2 cells and AO may be attributed to the fact that AO is a more physiologically relevant model with higher expression of TMPRSS2 and comparable expression levels of ACE2 as compared to bronchial explants, and our findings suggest that cell entry experiments of SARS-CoV-2 should not be using Vero E6-T2 cells, otherwise, interpretations may not reflect the situation in the human body.

A study found that the replication efficiency of BA.2.86 was similar to EG.5.1 in colon organoids^12^. However, we observed a slightly higher replication competence of EG.5.1 over BA.2.86 and JN.1 in human colon cells. The discrepancies may lie in the differences of cell populations in the colon organoids and colon cells. While the colon organoids have more goblet cells, non-differentiated colon cells mainly comprise colonocytes. By direct comparison, the WT virus replicated to the highest titres of all the tested variants, while BA.2.86 and JN.1 replicated less efficiently than EG.5.1 in colon cells. These findings are consistent with previous reports in colonoid-derived cells showing that Omicron infected less effectively than the WT strain in colon cells and induced limited expression of both type I and III interferons, which were comparable to wild-type virus and mock infections^15^. Partial reduction of BA.2.86 and JN.1 replication in colon cells by ACE2 inhibitor treatment suggests that factors other than ACE2 may contribute to the infection in colon cells which is in line with a report working with colonoid derived cells infected with wild-type and Omicron spike protein pseudovirus^16^.

Our study shows that BA.2.86 and JN.1 replicated efficiently in both proximal intestinal enteroids and colon cells, while EG.5.1 replicated efficiently in colon cells but has limited replication in proximal intestinal enteroids. In fact, many enteric viruses such as rotavirus and astrovirus, infect and replicate primarily in the small intestine causing viral shedding in stool leading to faecal-oral transmission^17,18^ which implying the importance of tropism in small intestine for faecal shedding and transmission. While clinical evidence shows that BA.2.86 and JN.1 infected patients have higher viral shedding in faecal samples when compared with patients infected with EG.5.1 and XBB.1.5^19^, our findings suggest that the tropism and replication competence of SARS-CoV-2 in proximal intestinal enteroids is more essential than in colon cells for virus spreading through the faecal samples. Furthermore, JN.1 induced relatively lower levels of pro-inflammatory cytokines than BA.2.86 in proximal intestinal enteroids, implying that there may not be severe digestive symptoms in the JN.1 infected patients. These observations are supported by the absence of severe digestive illnesses in patients with JN.1 infection^19^. The high replication competence in the intestine and mild or no digestive symptoms caused by JN.1 probably promote the spreading of this virus via the faecal route better than other variants.

Since EG.5.1, BA.2.86 and JN.1 had comparable cellular tropism in proximal intestinal enteroids, which cannot explain their differential replication competence. By using ACE2 inhibitor, the replication of JN.1 and BA.2.86 was reduced to 9% and 38% of vehicle treatment, respectively, showing that JN.1 infection is more dependent on ACE2 than BA.2.86 in proximal intestinal enteroids, similarly in colon cells, replication of JN.1 was more ACE2 and TMPRSS2 dependent than BA.2.86 and EG.5.1. We found that the ACE2 receptor, but not TMPRSS2 has a higher expression level in intestine enteroids than colon cells. BA.2.86 and JN.1 have a higher ACE2 binding affinity than EG.5.1 despite that JN.1 has a relatively lower ACE2 binding affinity than BA.2.86^1,13,20^. Therefore, high ACE2 binding affinity may contribute to the small intestine tropism. This is in line with the study showing that WT infection is associated with ACE2 expression in proximal intestinal organoid cultures^16^. These may also explain the higher prevalence of BA.2.86 and JN.1 observed over EG.5.1 in epidemiologic studies. Taken together, the elevated small intestine tropism of JN.1 and BA.2.86 may play an important role in their transmission and in becoming the predominant strains over EG.5.1.

EG.5.1 exhibits a higher effective reproduction number compared with XBB.1.5, XBB.1.16, and its parental lineage (XBB.1.9.2)^13^. This may be partly attributed to the higher immune evading ability of EG.5.1 than XBB.1.5 and XBB.1.9.2^21^, and EG.5.1 is more resistant to serum neutralisation from BQ or XBB breakthrough infection than XBB.1.16^3^. We also compared a number of BA.1 to BA.5 variants and XBB subvariants side-by-side with BA.5 in human bronchi and lung explants. Apart from the immune evasion, we found that like XBB.1.5, EG.5.1 replicated to higher titres in human bronchial and lung explants than XBB.1.16 and XBB.1.9.1, indicating that EG.5.1 has a growth advantage in the human respiratory tract, which contributes at least partially to the high prevalence over other XBB subvariants. Besides, EG.5.1 has similar lung tropism to BA.5 which is higher than that of BA.1 and BA.2 indicating that EG.5.1 gains replication fitness over BA.1 and BA.2. Reports showing that EG.5.1 and XBB.1.5 have similar growth kinetics and pathogenicity in hamsters and EG.5.1 is transmitted more efficiently between hamsters than BA.2^22^ which are in line with our findings on their bronchial and lung tropism.

The upsurge of BA.5 over BA.1 and BA.2 may be partly due to the reduced neutralisation titres against BA.5^23,24^. However, we provide additional information on their infection in lung explants. BA.5 replicating to higher titres than BA.2.12.1, CH.1.1, BA.2.86 and JN.1 indicating that BA.5 may cause more severe disease than BA.2 and its subvariants, which is in concordance to the higher hospitalisation rates of BA.5 infected patients than that of BA.2 infected individuals^12^. This may be attributed to the higher fusogenicity and enhanced ability to disrupt the respiratory epithelial and endothelial barriers, leading to the observed higher in-vivo pathogenicity of BA.5 than BA.1 and BA.2^23,25^.

In summary, we found that BA.5, BA.5.2.1, XBB.1.5, and EG.5.1 have relatively high replication competence in human bronchial and lung explants, which can promote viral spread and may lead to severe lung disease. Furthermore, although BA.2.86 and JN.1 replicated to lower titres than EG.5.1 in respiratory tissues, they showed higher replication competence in human proximal intestinal enteroids than EG.5.1, suggesting that these two viruses could be transmitted via the faecal-oral route. Moreover, we provide evidence demonstrating that ACE2 binding and TMPRSS2 are more essential for the replication of BA.2.86 and JN.1 in proximal intestinal enteroids than in colon cells. Besides, airway organoid is a more appropriate model than Vero E6-T2 cells for assessing SARS-CoV-2 host cell entry and proximal intestine tropism might be more relevant than colon tropism for the faecal route of virus dissemination.

SARS-CoV-2 variants can emerge from any previous lineages, for example, BA.2.86 and JN.1 are derived from BA.2, which is the precursor strain of the XBB lineage. Since immune protection in the community wanes quickly after vaccination or infection, characterization and surveillance of emerging SARS-CoV-2 variants are essential to safeguard public health and help better prepare for the epidemic or pandemic, especially, for those variants having high replication competence in the lower lung and transmitting efficiently between humans.

## Methods

### Viruses and cells

Vero-E6 cells with overexpressed TMPRSS2 (Vero-E6-T2) were provided by M. Takeda^26^ and were used for isolation and propagation of SARS-CoV-2 viruses^27^. Cells were maintained in DMEM (Gibco) supplemented with 10% foetal bovine serum (FBS; Gibco), 100 units/mL penicillin and 100 µg/mL streptomycin (Gibco). SARS-CoV-2 viruses, including the WT and Omicron lineages (BA.1, BA.2, BA.2.12.1, BA.4, BA.5, BA.5.2.1, XBB.1.5, XBB.1.9.1, XBB.1.16, CH.1.1, BQ.1.22, EG.5.1, BA.2.86 and JN.1) were used (Supplementary Table 1). The virus stocks were titrated in Vero-E6-T2 cells, aliquoted and stored frozen at -80 °C. All live-virus experiments were performed in a biosafety level 3 facility at the Li Ka Shing Faculty of Medicine, The University of Hong Kong.

### Ex vivo cultures and infection of human respiratory tract

Human non-tumour bronchus (26 donors) and lung explants (26 donors) were obtained from patients aged 45-81 years undergoing elective surgery in the Department of Surgery at Queen Mary Hospital from June 2022 to Jan 2026 and were removed as part of the clinical care but surplus for routine diagnostic requirements, as described previously^28^. Bronchus and lung explants were fragmented and infected with 5 x 10^4^ pfu at 37 °C for 1 h. The infected tissue explants were washed three times, placed in fresh medium and incubated at 37 °C with 5% CO_2_. Mock-infected tissues were used as controls. Culture supernatants were collected at the indicated times to assess infectious viral titres. Tissues were harvested at endpoints and fixed in 10% formalin for immunostaining. Ethics approval of the use of human tissues was granted by the institutional review board of The University of Hong Kong and the Hospital Authority (Hong Kong West) (IRB approval no: UW 20-862 and UW20-863). All participants provided written informed consent. The information of tissue donors is listed in the Supplementary Table 2.

### Human airway organoid culture and infection

Human airway organoids were established from human lung explants as previously described^29^, and were differentiated into 2-dimensional model as described elsewhere^30^ with slight modifications. In brief, airway organoids were dissociated into single cells by digestion using TrypLE Express Enzyme (Gibco) at 37 °C for 10 min, then sheared with 25-gauge needles and filtered through 40 μm cell strainers. 1.5 x 10^5^ cells were seeded onto 0.4 μm pore size Transwell inserts (Corning) pre-coated with rat tail collagen I (Corning). The cells were cultured in a mixture of airway organoid growth medium and PneumaCult-ALI complete base medium (Stemcell Technologies) at a ratio of 1:1 at 37 °C for 3-4 days. After reaching confluency, the 2-dimensional transwell cultures were cultured at air-liquid interface (ALI) in PneumaCult-ALI Maintenance Medium (Stemcell Technologies) and used for infection after 2 weeks of differentiation. Cells at the apical side were infected with viruses at a multiplicity of infection (MOI) of 0.1 at 37 °C for 1 h, washed with PBS and cultured at ALI in the same growth medium. At 1, 24 and 48 hpi, culture supernatants from the apical side of transwells were collected for viral titres determination. The transwell cultures were harvested at endpoint in RLT Buffer or fixed in 10% formalin.

### Human proximal intestinal enteroid culture and infection

Human proximal intestinal enteroids were established from normal regions of human duodenal tissues as previously described^31–33^ with modifications. Proximal intestinal enteroids were derived through isolation of intestinal crypts, grown in 3-dimensional cultures in Matrigel (Corning) and were maintained in an in-house Complete medium with growth factors (CMGF+) supplemented with 10 µM Rho-associated protein kinase inhibitor Y-27632 (Stemcell Technologies)^33^. Proximal intestinal enteroids were differentiated by incubating in Advanced DMEM/F12 medium (Gibco) supplemented with IntestiCult Organoid Growth Medium (OGM) Human Component A (Stemcell Technologies) and GlutaMAX-I (Gibco) for 3 to 4 days, and were dissociated using Gentle Cell Dissociation Reagent (Stemcell Technologies) immediately before infections. Proximal intestinal enteroids were infected with viruses at 10^6^ pfu/mL at 37 °C for 1 h, washed with Advanced DMEM/F12 and cultured in Matrigel with the differentiation medium. Culture supernatants were collected at 1, 24, 48 and 72 hpi. Infected proximal intestinal enteroids were lysed in RLT Buffer or fixed in 10% formalin at endpoints. Ethics approval of the use of human tissues was granted by the institutional review board of The University of Hong Kong and the Hospital Authority (Hong Kong West) (IRB approval no: UW 20-575). All participants provided written informed consent. The information of tissue donors is listed in the Supplementary Table 2.

### Caco-2 cell culture and infection

Human colorectal adenocarcinoma Caco-2 (colon) cells (ATCC) were cultured on vessel surface coated with rat tail collagen I (Corning), and maintained in Minimum Essential Medium (MEM, Gibco) supplemented with 10% foetal bovine serum (FBS; Gibco), 100 units/mL penicillin and 100 µg/mL streptomycin (Gibco). Cells were split and sub-cultured when reaching 80–90% confluency. 1 × 10^5^ cells were seeded and cultured for 2 days before virus infection. Infection was performed at MOI 0.1, and culture supernatants were collected at 1, 24 and 48 hpi for viral titres detection. Cells were lysed in RLT Buffer for mRNA expression analysis.

### Viral titration

96-well tissue culture plates of confluent Vero-E6-T2 cells were prepared one day before the virus titration by 50% tissue culture infectious dose (TCID_50_) assay. Cells were seeded with 2% FBS/DMEM. Culture supernatants were serially diluted from 0.5-log to 7-log and added onto the cell plates in quadruplicate. The cell plates were observed for CPE after 3 days. The endpoints of viral dilution leading to CPE in 50% of the inoculated wells were used to calculate viral titres using the Spearman-Kärber method. Area under the curve (AUC) was calculated by integrating the infectious virus titres at 24–72 hpi.

### RNA extraction and qPCR

Total RNA was extracted from cell lysates using MiniBEST Universal RNA Extraction Kit (TaKaRa Bio). RNA was reverse transcribed with PrimeScript RT Reagent Kit (TaKaRa Bio). mRNA expressions of viral and cytokine genes were quantified using SYBR Premix Ex Taq II (TaKaRa Bio) and measured with ViiA7 Real-Time PCR System (Applied Biosystems). The gene expression levels were quantified with the respective standards and normalised with β-actin, as previously described^27^. The sequences of the primers are provided in Supplementary Table 3.

### Drug treatment

To assess the replication dependency on specific host factors, TMPRSS2 inhibitor, camostat mesylate (CAM)(Sigma-Aldrich) at 60 µM, cathepsins inhibitor, E64d (Sigma-Aldrich) at 60 µM, ACE2 inhibitor, Ouabain (Oua)(Sigma-Aldrich) at 75 nM was added to the cultures 1 h before, during and after infection. The vehicle was used as a negative control. Viral titres in culture supernatants were determined using TCID_50_ assay.

### Immunohistochemistry staining

Infected ex vivo and organoids were fixed with 10% formalin for at least 3 days before being embedded in paraffin blocks and sectioned into 4 µm thickness onto glass slides. For immunohistochemistry, the tissue sections were microwaved in 10 mM citrate buffer for 15 min for antigen retrieval. Endogenous peroxidase activity was stopped by quenching the tissue sections with 3% H_2_O_2_ for 20 min. The slides were blocked with 10% normal horse serum at room temperature and then incubated with primary antibodies: anti-SARS-CoV-2 nucleocapsid (N) protein (40143-T62, Sino Biological) for 90 min at room temperature, followed by horseradish peroxidase-conjugated secondary antibodies (MP-7401-50, Vector Laboratory). The section stainings were developed using NovaRED Substrate Kit (SK-4800, Vector Laboratory) and counterstained for cell nuclei with Mayer’s Hematoxylin. The sections were imaged using the ECLIPSE Ti-S microscope (Nikon Instruments).

### Immunostaining of human respiratory tissues

To characterize the SARS-CoV-2-infected cells, the respiratory tract tissues (lung and bronchus) were subjected to double-antibody immunofluorescence staining of SARS-CoV-2 spike antibody or SARS-CoV-2 Nucleocapsid antibody (N) with different lung and bronchus markers as follows. The fixed tissues embedded in paraffin block were sectioned and microwaved in 10 mM citrate buffer pH 6.0 for 20 mins and 5 mins to expose antigens, respectively. The sections were blocked with 10% normal goat serum for 10 min at room temperature (RT) and then incubated with either SARS-CoV-2 spike (99423, Cell Signalling) or SARS-CoV-2 N (40143, Sino Biological) antibodies for 90 min at RT followed by alkaline phosphatase (AP) conjugated anti-rabbit (MP-5401-15, Vector Laboratory) or anti-mouse antibodies (MP-5402-15, Vector Laboratory) for 60 min at RT. The sections were developed using Vector® Red (VR) Substrate Kit (SK-5100, Vector Laboratory). The sections were then microwaved, incubated with SCGB1A1/CC10 (Protein-tech), p63-alpha (13109, Cell Signalling), acetylated α Tubulin (SC-23950, Santa Cruz), MUC5AC (MA5-12178, Invitrogen), HT2-280 (TB-27AHT2-280, Terrace Biotech), AGER (HPA069474, Sigma-Aldrich), CD68 (76437, Cell Signalling), ACE2 (ab108252, Abcam) or TMPRSS2 (ab109131, Abcam) for 75 to 90 min at RT followed by donkey anti rabbit-AF594 (A21207, Invitrogen) or donkey anti mouse-AF488 (A32766, Invitrogen) for 60 min at RT. The cell nuclei were counterstained with DAPI (564907, BD Bioscience) (blue). The sections were mounted with fluorescent mounting medium (Dako) and imaged using a Nikon Eclipse Ti-S microscope.

### Immunofluorescence staining of human proximal intestinal enteroids

Human proximal intestinal enteroids were fixed with 10% formalin for at least 3 days and the fixed organoids were embedded in paraffin blocks. To characterize the SARS-CoV-2-infected cells, double-antibody immunofluorescence staining of SARS-CoV-2 N antibody with different intestinal markers was performed. The fixed organoids embedded in a paraffin block were sectioned and microwaved in a 10mM citrate buffer, pH 6.0, for 5min to expose antigens, blocked with 10% normal goat serum for 10min at room temperature (RT). The sections were then incubated with SARS-CoV-2 N antibody along with one of the following antibodies: FABP1 (HPA028275, Abcam), Mucin-2 (ab90007, Abcam), Lysozyme C (A0099, Dako), or ACE2 (ab108252, Abcam) or TMPRSS2 (ab109131, Abcam) for 60 min at RT followed by donkey anti-mouse Alexa Fluor 594 and donkey anti-rabbit Alexa Fluor 488 for 60 min at RT in dark. The cell nuclei were counterstained with DAPI (blue). The sections were mounted with fluorescent mounting medium (Dako), and images were taken using a Nikon Eclipse Ti-S microscope.

### Statistical analysis

Experiments with human ex vivo tissues were performed using at least six donors, and organoid cultures were performed in three donors each in duplicate or triplicate. Results were presented as the mean values ± standard deviation (SD). Statistical analyses were performed either using one-way ANOVA followed by a Tukey’s multiple-comparison test or two-way ANOVA followed by a Tukey’s multiple-comparison test using GraphPad Prism version 10 (GraphPad Software), with the differences considered to be significant at p values of less than 0.05.

### Reporting summary

Further information on research design is available in the Nature Portfolio Reporting Summary linked to this article.

## Supplementary information

Supplementary information

Reporting summary

Transparent Peer Review file

## Source data

Source Data

## Data Availability

Source data are provided with this paper.
