# Supplementary material for: SARS-CoV-2 Omicron BA.2.86 and JN.1 expand tropism in human proximal intestinal epithelium
Source: Nat Commun. 2026 Jun 5;17:7187. doi: 10.1038/s41467-026-74111-y (PMC13396732; doi:10.1038/s41467-026-74111-y)
Supplement: Supplementary file 1 — Supplementary information [file 41467_2026_74111_MOESM1_ESM.pdf]

SARS-CoV-2 Omicron BA.2.86 and JN.1 expand tropism in human proximal intestinal epithelium

Kenrie PY Hui, John CW Ho, Ka-Chun Ng, Jenny CM Chan, Taylor WC Ho, Rachel HH Ching, Leo LM Poon, Malik Peiris, John M Nicholls and Michael CW Chan

Supplementary Information

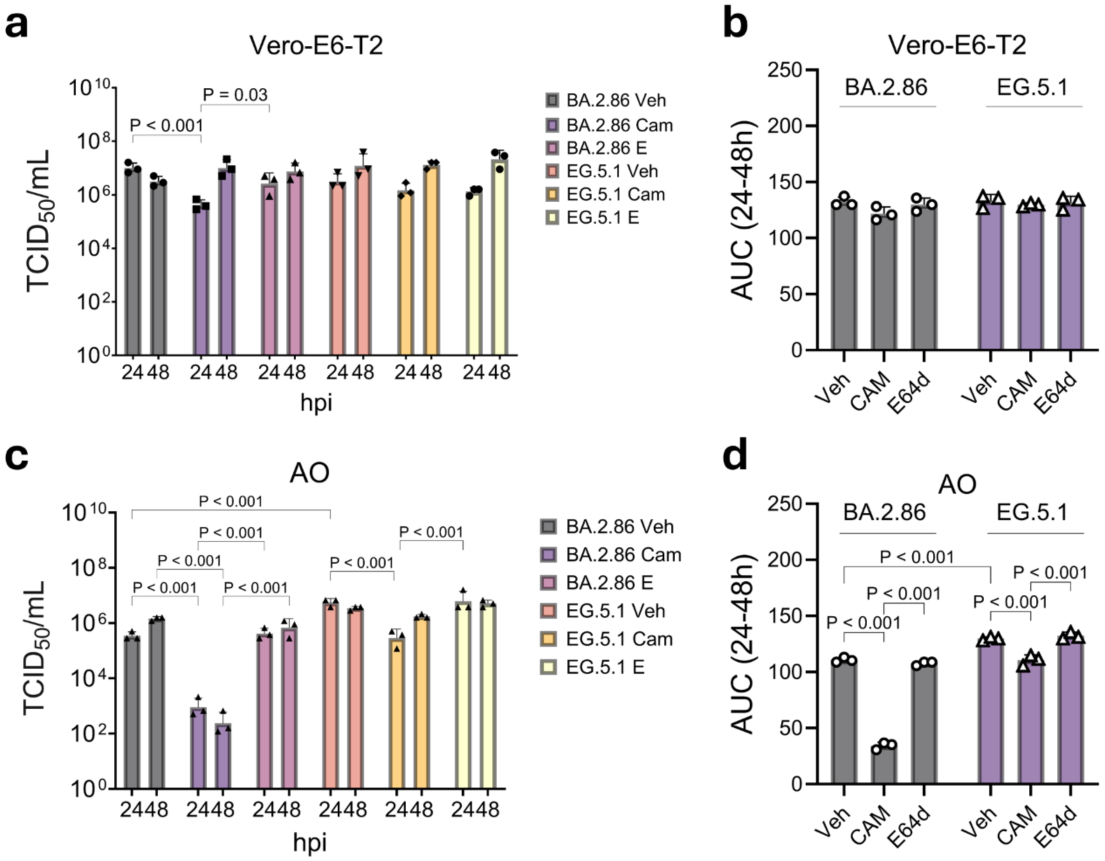

Supplementary Figure 1. Dependence on TMPRSS2 and cathepsins of host cell entry of BA.2.86 and EG.5.1 in Vero-E6-T2 cells and human airway organoids. Replication of BA.2.86 and EG.5.1 in the presence of camostat mesylate (CAM), E64d or vehicle (Veh) in (a and b) Vero-E6-T2 cells and (c and d) human airway organoids. Viral titres in culture supernatants were shown in a and c. Viral titres at 1 hpi were undetectable and are not shown. The horizontal dotted line denotes the limit of detection in the $TCID_{50}$ assay. (b and d) Viral titres from a and c are depicted as area under the curve (AUC). Bar-charts show the mean value ± standard deviation (SD)(n=3). a to d, Statistical significance was calculated using two-way ANOVA followed by Tukey's multiple comparisons test. *P*<0.05 was considered to be statistically significant, and exact *P* values are presented.

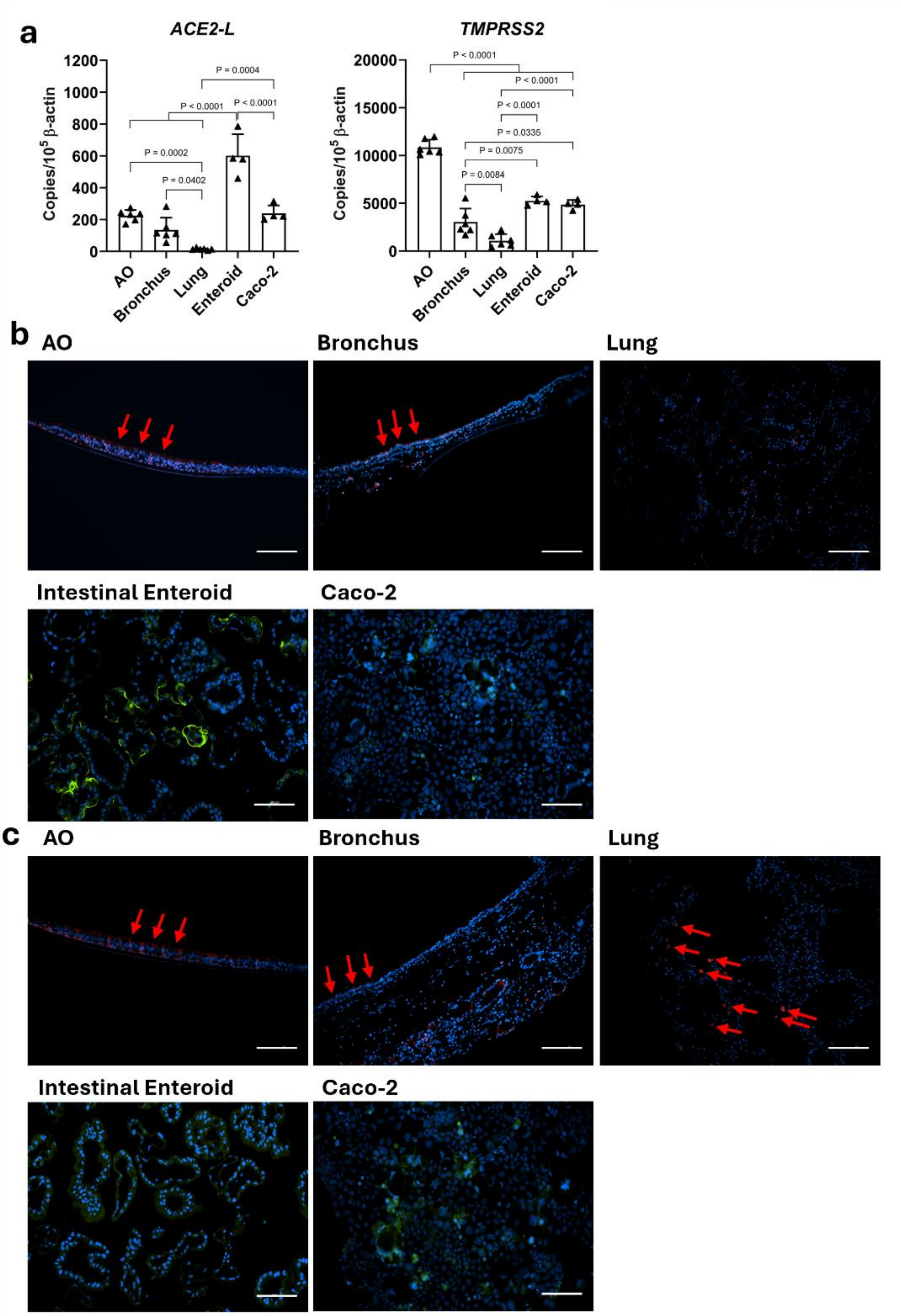

Supplementary Figure 2. Expression of ACE2-L and TMPRSS2 in airway organoids, bronchus, lung explants, proximal intestinal enteroids and colon cells. mRNA expression of (a) *ACE2-L* and *TMPRSS2* in airway organoids (AO) (n=6), bronchus (n=6), lung explants

(n=6), proximal intestinal enteroids (n=4) and colon cells (n=4) is shown. Data are the mean ± SD. Statistical analysis was performed using one-way ANOVA followed by Tukey's multiple comparisons test. $P<0.05$ was considered to be statistically significant, and exact P values are presented. Immunofluorescence staining of (b) ACE2-L and (c) TMPRSS2 was performed in the above-mentioned models. In AO, bronchus and lung explants, ACE2-L and TMPRSS2 are shown in red and nuclei are in blue. Red arrows indicate the staining of ACE2-L and TMPRSS2. In proximal intestinal enteroids and colon cells, ACE2-L and TMPRSS2 are shown in green and nuclei are in blue. The images are representatives of two individual donors. Scale bars, 100 μm.

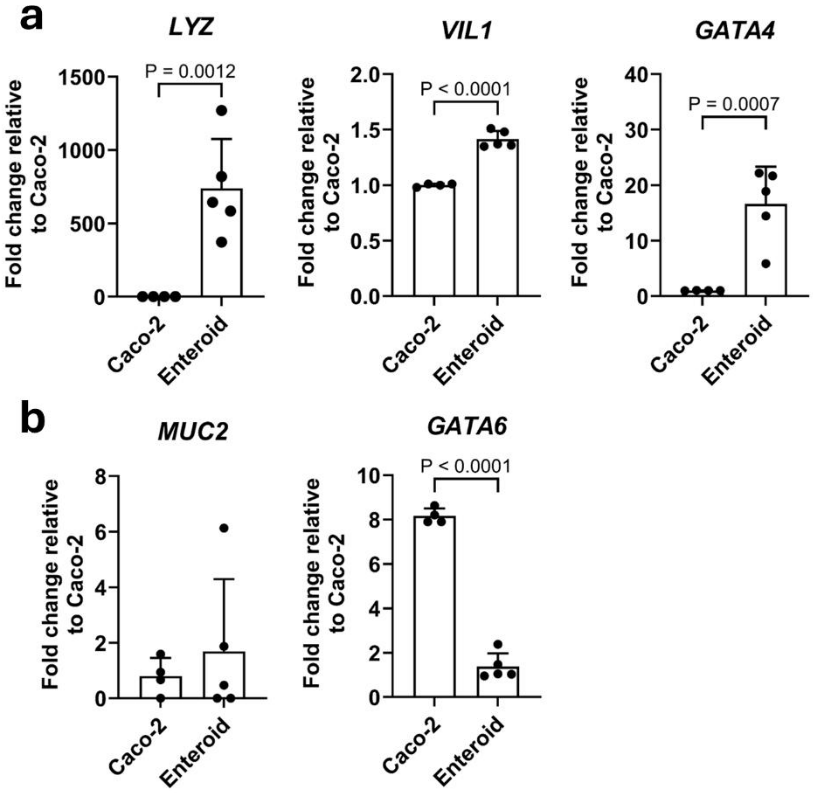

Supplementary Figure 3. Expression of cellular markers of intestinal epithelium and colon in proximal intestinal enteroids and colon cells. mRNA expression of (a) small intestine cell markers *lyzozme (LYZ), villin 1 (VIL1), GATA binding protein 4 (GATA4)* and (b) colon cell markers *mucin-2 (MUC2) and GATA binding protein 6 (GATA6)* in uninfected proximal intestinal enteroids (n=5) and colon cells (n=4) were shown. Data are the mean ± SD. Statistical analysis was performed using one-way ANOVA followed by Tukey's multiple comparisons test. $P<0.05$ was considered to be statistically significant, and exact P values are presented.

Supplementary Table 1. Information of virus isolates

Virus	Strain name	Accession ID
WT	hCoV-19/Hong Kong/VM20001061/2020	EPI_ISL_408975
BA.1	hCoV-19/Hong Kong/VM21044713_WHP5047-S5/2021	EPI_ISL_6716902
BA.2	hCoV-19/Hong Kong/VOC-588-P3-S18-iseq/2022	EPI_ISL_11222954
BA.4	hCoV-19/USA/MD-HP30386_HKUP3/2022	OP099836.1
BA.5	SARS-CoV-2/human/USA/COR-22-063113/2022	ON972631.1
BA.2.12.1	SARS-CoV-2/human/USA/COR-22-062161/2022	OP589872.1
BA.5.2.1	hCoV-19/Hong Kong/HKUSPH_WHP7830P2/2022	EPI_ISL_19205705
XBB.1.5	hCoV-19/USA/MD-HP40900/2022	EPI_ISL_16026423
CH.1.1	hCoV-19/Hong Kong/HKUSPH_VOC1424FP3/2022	EPI_ISL_19239240
BQ.1.22	hCoV-19/Hong Kong/HKUSPH_VOC1464P2/2022	EPI_ISL_19239241
XBB.1.16	hCoV-19/Hong Kong/HKUSPH_VOC1933P3/2023	EPI_ISL_18604375
XBB.1.9.1	hCoV-19/Hong Kong/HKUSPH_VOC1636P3/2023	EPI_ISL_18604376
EG.5.1	hCoV-19/Hong Kong/HKUSPH_VOC2249P3/2023	EPI_ISL_18604378
BA.2.86	hCoV-19/Hong Kong/HKUSPH_LRS0548P3/2023	EPI_ISL_18604485
JN.1	hCOV-19/Hong Kong/HKUSPH_VOC2401P2/2023	EPI_ISL_18888405

Supplementary Table 2. Information of tissue donors

PATIENT	TISSUE USED	AGE	GENDER
**VIRUSES: WT, BA.1, BA.2, BA.2.12.1, BA.4, BA.5, BA.5.2.1**			
**1**	Bronchus and Lung	68	M
**2**	Bronchus and Lung	47	M
**3**	Bronchus and Lung	70	M
**4**	Bronchus and Lung	75	M
**5**	Bronchus and Lung	71	M
**6**	Bronchus and Lung	78	M
**7**	Bronchus	59	F
**VIRUSES: BA.5, XBB.1.5, CH.1.1, BQ.1.22, XBB.1.16, XBB.1.9.1**			
**8**	Bronchus and Lung	45	F
**9**	Bronchus and Lung	71	M
**10**	Bronchus and Lung	73	M
**11**	Bronchus and Lung	77	F
**12**	Bronchus and Lung	61	M
**13**	Bronchus and Lung	76	M
**14**	Lung	64	M
**VIRUSES: BA.5, XBB.1.16, XBB.1.9.1, EG.5.1, BA.2.86, JN.1**			
**15**	Bronchus and Lung	55	F
**16**	Bronchus and Lung	72	M
**17**	Bronchus and Lung	81	M
**18**	Bronchus and Lung	56	F
**19**	Bronchus and Lung	65	M
**20**	Bronchus and Lung	68	M
**VIRUSES: WT, BA.1, XBB.1.5, EG.5.1, BA.2.86, JN.1**			
**21**	Bronchus and Lung	65	M
**22**	Bronchus and Lung	61	M
**23**	Bronchus and Lung	76	M
**24**	Bronchus and Lung	73	M
**25**	Bronchus and Lung	71	M
**26**	Bronchus and Lung	69	M
**VIRUSES: WT, BA.1, XBB.1.5, EG.5.1, BA.2.86, JN.1**			
**1**	Small intestine	87	F
**2**	Small intestine	74	F
**3**	Small intestine	83	F

Supplementary Table 3. The nucleotide sequences of primers used for quantitative PCR.

Gene	Primer sequence (5'-3')
ACTB (β-actin)	F: TGGATCAGCAAGCAGGAGTATG<br>R: GCATTTGCGGTGGACGAT
SARS-CoV-2 *ORF1b*	F: TGGGGYTTTACRGGTAACCT<br>R: AACRCGCTTAACAAAGCACTC
IFNA1 (IFN-α)	F: AGAAGGCTCCAGCCATCTCTGT<br>R: TGCTGGTAGAGTTCGGTGCAGA
IFNB1 (IFN-β)	F: CAACTTGCTTGGATTCCTACAAAG<br>R: TGCCACAGGAGCTTCTGACA
IFNL1 (IFN-λ1)	F: GCCCCCAAAAAGGAGTCCG<br>R: AGGTTCCCATCGGCCACATA
IFNL2/3 (IFN-λ2/3)	F: TTTAAGAGGGCCAAAGATGC<br>R: TGGGCTGAGGCTGGATACAG
CXCL10 (IP-10)	F: ATTATTCCTGCAAGCCAATTTTG<br>R: TCACCCTTCTTTTTCATTGTAGCA
ISG15	F: CAAATGCGACGAACCTCTGA<br>R: CCGCTCACTTGCTGCTTCA
IFIH1 (MDA5)	F: TCACAAGTTGATGGTCCTCAAGT<br>R: CCTTCTCCAGATTTGGCTGAAC
TNF (TNF-α)	F: GCAGGTCTACTTTGGGATCATTG<br>R: GCGTTTGGGAAGGTTGGA
ACE2-L	F: CAAGAGCAAACGGTTGAACAC<br>R: CCAGAGCCTCTCATTGTAGTCT
TMPRSS2	F: CAAGTGCTCCAACTCTGGGAT<br>R: AACACACCGATTCTCGTCCTC
LYZ	F: AAAACCCCAGGAGCAGTTAAT<br>R: CAACCCTCTTTGCACAAGCT
VIL1	F: AGCCAGATCACTGCTGAGGT<br>R: TGGACAGGTGTTCCTCCTTC
GATA4	F: CGACACCCCAATCTCGATATG<br>R: GTTGCACAGATAGTGACCCGT
MUC2	F: ACTCCAACATCTCCGTGTCC<br>R: AGCCACACTTGTCTGCAGTG
GATA6	F: GCCACTACCTGTGCAACGCCT<br>R: CAATCCAAGCCGCCGTGATGAA
