# Supplementary material for: SARS-CoV-2 Omicron BA.2.86 and JN.1 expand tropism in human proximal intestinal epithelium
Source: Nat Commun. 2026 Jun 5;17:7187. doi: 10.1038/s41467-026-74111-y (PMC13396732; doi:10.1038/s41467-026-74111-y)
Supplement: Supplementary file 2 — Reporting summary [file 41467_2026_74111_MOESM2_ESM.pdf]

nature portfolio

Corresponding author(s): Michael CW Chan

Last updated by author(s): May 20, 2026

Reporting Summary

Nature Portfolio wishes to improve the reproducibility of the work that we publish. This form provides structure for consistency and transparency in reporting. For further information on Nature Portfolio policies, see our Editorial Policies and the Editorial Policy Checklist.

Statistics

For all statistical analyses, confirm that the following items are present in the figure legend, table legend, main text, or Methods section.

n/a	Confirmed	
☐	☒	The exact sample size (*n*) for each experimental group/condition, given as a discrete number and unit of measurement
☐	☒	A statement on whether measurements were taken from distinct samples or whether the same sample was measured repeatedly
☐	☒	The statistical test(s) used AND whether they are one- or two-sided<br>*Only common tests should be described solely by name; describe more complex techniques in the Methods section.*
☐	☒	A description of all covariates tested
☒	☐	A description of any assumptions or corrections, such as tests of normality and adjustment for multiple comparisons
☐	☒	A full description of the statistical parameters including central tendency (e.g. means) or other basic estimates (e.g. regression coefficient) AND variation (e.g. standard deviation) or associated estimates of uncertainty (e.g. confidence intervals)
☐	☒	For null hypothesis testing, the test statistic (e.g. *F*, *t*, *r*) with confidence intervals, effect sizes, degrees of freedom and *P* value noted<br>*Give P values as exact values whenever suitable.*
☒	☐	For Bayesian analysis, information on the choice of priors and Markov chain Monte Carlo settings
☒	☐	For hierarchical and complex designs, identification of the appropriate level for tests and full reporting of outcomes
☒	☐	Estimates of effect sizes (e.g. Cohen's *d*, Pearson's *r*), indicating how they were calculated

Our web collection on statistics for biologists contains articles on many of the points above.

Software and code

Policy information about availability of computer code

Data collection	GraphPad Prism v10.2.3 was used to produce figures.
Data analysis	GraphPad Prism v10.2.3 was used for statistical analses.

For manuscripts utilizing custom algorithms or software that are central to the research but not yet described in published literature, software must be made available to editors and reviewers. We strongly encourage code deposition in a community repository (e.g. GitHub). See the Nature Portfolio guidelines for submitting code & software for further information.

Data

Policy information about availability of data

All manuscripts must include a data availability statement. This statement should provide the following information, where applicable:

- Accession codes, unique identifiers, or web links for publicly available datasets
- A description of any restrictions on data availability
- For clinical datasets or third party data, please ensure that the statement adheres to our policy

All data generated or analysed during this study are included in this published article (and its supplementary information files). Source data are provided with this paper.

Research involving human participants, their data, or biological material

Policy information about studies with human participants or human data. See also policy information about sex, gender (identity/presentation), and sexual orientation and race, ethnicity and racism.

Reporting on sex and gender	This study does not involve research on human populations defined by attributes of sex or gender.
Reporting on race, ethnicity, or other socially relevant groupings	This study does not involve research on human populations defined by attributes of race, ethnicity, national or social origin.
Population characteristics	Patient demographic data was provided in the supplementary information.
Recruitment	Patients undergone elective surgery were recruited with written informed consents at Hong Kong Queen Mary Hospital.
Ethics oversight	Ethics approval of the use of human tissues was granted by the institutional review board of The University of Hong Kong and the Hospital Authority (Hong Kong West).

Note that full information on the approval of the study protocol must also be provided in the manuscript.

Field-specific reporting

Please select the one below that is the best fit for your research. If you are not sure, read the appropriate sections before making your selection.

☒ Life sciences ☐ Behavioural & social sciences ☐ Ecological, evolutionary & environmental sciences

For a reference copy of the document with all sections, see nature.com/documents/nr-reporting-summary-flat.pdf

Life sciences study design

All studies must disclose on these points even when the disclosure is negative.

Sample size	The sample size was chosen based on the magnitude and consistency of measurable differences between groups to satisfy the thresholds for statistical power. For in vitro models, experiments were performed at least three times to provide n= or >3. The sample number has been indicated in the figure legends. The sample number has been indicated in the figure legend and methods.
Data exclusions	There is no data exclusions.
Replication	In vitro experiments were done in technical duplicates or triplicates and each experiment was repeated.
Randomization	Not applicable as this is not an intervention study.
Blinding	The experiments and sample processing were performed by different individuals. Sample identities were blinded during data analysis.

Reporting for specific materials, systems and methods

We require information from authors about some types of materials, experimental systems and methods used in many studies. Here, indicate whether each material, system or method listed is relevant to your study. If you are not sure if a list item applies to your research, read the appropriate section before selecting a response.

Materials & experimental systems

n/a	Involved in the study	
☐	☒	Antibodies
☐	☒	Eukaryotic cell lines
☒	☐	Palaeontology and archaeology
☒	☐	Animals and other organisms
☒	☐	Clinical data
☒	☐	Dual use research of concern
☒	☐	Plants

Methods

n/a	Involved in the study	
☒	☐	ChIP-seq
☒	☐	Flow cytometry
☒	☐	MRI-based neuroimaging

Antibodies

Antibodies used	1. SARS-CoV/SARS-CoV-2 Nucleocapsid Antibody, Rabbit PAb, Sino Biological, 40143-T62 1:2000<br>2. SARS-CoV/SARS-CoV-2 Nucleocapsid Antibody, Mouse MAb, Sino Biological, 40143-MM05 1:1000

3. SARS-CoV-2 Spike Protein (S1) (E5S3V), Rabbit Monoclonal Antibody, Cell Signaling, E5S3V 1:200
4. Anti-FABP1 antibody, Rabbit PAb, Abcam, HPA028275 1:300
5. Anti-MUC2 antibody, Rabbit PAb, Abcam, ab90007 1:200
6. Lysozyme EC 3.2.1.17, Rabbit PAb, Dako, A0099 1:2000
7. Anti-ACE2 antibody, Rabbit MAb, Abcam, ab108252 1:200
8. Anti-TMPRSS2 antibody, Rabbit MAb, Abcam, ab109131 1:50
9. Acetylated α Tubulin Antibody, Mouse MAb, SC-23950, Santa Cruz 1:100
10. MUC5AC antibody, Mouse MAb, MA5-12178, Invitrogen 1:50
11. Uteroglobin/CC10 Polyclonal antibody, Rabbit PAb, 10490-1-AP, Proteintech 1:100
12. p63-alpha (D2K8X), Rabbit MAb, 13109, Cell Signaling 1:50
13. Anti HT2-280, Mouse MAb, TB-27AHT2-280, Terrace Biotech 1:150
14. Anti-AGER antibody, Rabbit PAb, HPA069474, Sigma-Aldrich 1:50
15. CD68 (D4B9C), Rabbit MAb, 76437, Cell Signaling 1:500
16. Donkey anti-Mouse IgG (H+L) Cross-Adsorbed Secondary Antibody, Alexa Fluor Plus 488 #A32766, Invitrogen 1:100
17. Donkey anti-Rabbit IgG (H+L) Cross-Adsorbed Secondary Antibody, Alexa Fluor 594 #A21207, Invitrogen 1:100
18. BD Pharmingen™ DAPI Solution 564907, BD Bioscience 1ug/ml
19. ImmPRESS Horse anti-Mouse-HRP Polymer, Vectorlab, MP-7402
20. Vector NovaRed substrate kit (HRP), Vectorlab, SK-4800
21. ImmPress HRP Horse Anti-Rabbit IgG Kit, Vector Laboratory, MP-7401-50
22. ImmPress AP Horse Anti-Rabbit IgG Kit, Vector Laboratory, MP-5401-15
23. ImmPress AP Horse Anti-Mouse IgG Kit, Vector Laboratory, MP-5402-15

Validation

Validation:
1. SARS-CoV/SARS-CoV-2 Nucleocapsid Antibody, Rabbit PAb, Sino Biological, 40143-T62
• Validated applications: ELISA and WB
2. Immunocytochemistry SARS-CoV/SARS-CoV-2 Nucleocapsid Antibody Monoclonal Mouse 40143-MM05 [IgG1 Clone #05] Sino Biological
• Validated applications: ELISA, WB, IHC-P and Flow
3. SARS-CoV-2 Spike Protein (S1) (E5S3V) Rabbit Monoclonal Antibody #99423, Cell Signaling
• Validated applications: WB, IHC and IF
4. Anti-FABP1 antibody produced in rabbit, HPA028275, Sigma-Aldrich
• Validated applications: IHC-P
5. Immunocytochemistry/Immunofluorescence Anti-MUC2 antibody Polyclonal Rabbit ab90007 Abcam
• Validated applications: IHC-P and ICC/IF
6. Immunocytochemistry/Immunofluorescence Lysozyme EC 3.2.1.17 Polyclonal Rabbit A0099 Abcam
• Validated applications: IHC, IHC-IF, WB and IF
7. Anti-ACE2 antibody monoclonal rabbit [EPR4435(2)], ab108252, Abcam
• Validated applications: WB, IP, IHC-P and ELISA
8. Anti-TMPRSS2 antibody monoclonal rabbit [EPR3862], ab109131, Abcam
• Validated applications: WB and IHC-P
9. Acetylated α Tubulin Antibody, Mouse MAb, SC-23950, Santa Cruz
• Validated applications: WB, IHC-P and IF
10. MUC5AC antibody, Mouse MAb, MA5-12178, Invitrogen
• Validated applications: IHC-P and ICC/IF
11. Uteroglobin/CC10 Polyclonal antibody, Rabbit PAb, 10490-1-AP, Proteintech
• Validated applications: IHC, IF-P and Flow
12. p63-alpha (D2K8X) Rabbit Monoclonal Antibody, 13109, Cell Signaling
• Validated applications: WB, IP, IF, Flow, ChIP and C&R
13. Anti HT2-280, Mouse MAb, TB-27AHT2-280, terracebiotech
• Validated applications: IHC
14. Anti-AGER antibody, Rabbit PAb, HPA069474, Sigma-Aldrich
• Validated applications: IF and IHC
15. CD68 (D4B9C) Rabbit Monoclonal Antibody, 76437, Cell Signaling
• Validated applications: IHC, IF and Flow
16. Immunocytochemistry Donkey anti-Mouse IgG (H+L) Cross-Adsorbed Secondary Antibody, Alexa Fluor Plus 488 # A32766, Invitrogen
• Validated applications: WB and ICC/IF
17. Immunocytochemistry Donkey anti-Rabbit IgG (H+L) Cross-Adsorbed Secondary Antibody, Alexa Fluor 594 # A21207, Invitrogen
• Validated applications: IHC(F), ICC/IF and Flow
18. Immunocytochemistry BD Pharmingen™ DAPI Solution 564907 BD Bioscience
• Validated applications: IF and Flow

Eukaryotic cell lines

Policy information about cell lines and Sex and Gender in Research

Cell line source(s)

Vero E6-TMPRSS2 cells were a kind gift from Dr. Makoto Takeda (Director, Department of Virology 3, National Institute of Infectious Diseases, Tokyo, Japan)

Authentication

None of the cell lines used were authenticated.

Mycoplasma contamination | All cell lines used were tested by PCR and were mycoplasma free.

Commonly misidentified lines (See ICLAC register) | No commonly misidentified lines were used in this study.

Plants

Seed stocks

Report on the source of all seed stocks or other plant material used. If applicable, state the seed stock centre and catalogue number. If plant specimens were collected from the field, describe the collection location, date and sampling procedures.

Novel plant genotypes

Describe the methods by which all novel plant genotypes were produced. This includes those generated by transgenic approaches, gene editing, chemical/radiation-based mutagenesis and hybridization. For transgenic lines, describe the transformation method, the number of independent lines analyzed and the generation upon which experiments were performed. For gene-edited lines, describe the editor used, the endogenous sequence targeted for editing, the targeting guide RNA sequence (if applicable) and how the editor was applied.

Authentication

Describe any authentication procedures for each seed stock used or novel genotype generated. Describe any experiments used to assess the effect of a mutation and, where applicable, how potential secondary effects (e.g. second site T-DNA insertions, mosiacism, off-target gene editing) were examined.
