# Supplementary material for: SARS-CoV-2 Omicron BA.2.86 and JN.1 expand tropism in human proximal intestinal epithelium
Source: Nat Commun. 2026 Jun 5;17:7187. doi: 10.1038/s41467-026-74111-y (PMC13396732; doi:10.1038/s41467-026-74111-y)
Supplement: Supplementary file 3 — Transparent Peer Review file [file 41467_2026_74111_MOESM3_ESM.pdf]

nature portfolio

Peer Review File

SARS-CoV-2 Omicron BA.2.86 and JN.1 expand tropism in human proximal intestinal epithelium

Corresponding Author: Professor Michael C. W. Chan

This file contains all reviewer reports in order by version, followed by all author rebuttals in order by version.

Version 0:

Reviewer comments:

Reviewer #1

(Remarks to the Author)
The manuscript 'SARS-CoV-2 Omicron BA.2.86 & JN.1 shifting tropism from airway to intestine' by Hui et al. investigates the replication competence and tropism of different SARS-CoV-2 Omicron sublines, including BA.1 to JN.1, using a range of experimental models such as human bronchial and lung explants, airway organoids and intestinal enteroids. The authors aim to assess differences in cell entry pathways, extrapulmonary infection potential and immune responses between these variants.
The study shows that BA.5, XBB.1.5 and EG.5.1 replicate to higher titers in bronchial and lung tissue compared to BA.2.86 and JN.1. In addition, BA.2.86 shows increased replication in intestinal enteroids, with higher ACE2 expression in the intestinal epithelium, while EG.5.1 shows cell entry via two pathways, contributing to its pulmonary tropism and pathogenicity. The authors conclude that respiratory organoids and intestinal enteroids provide more clinically relevant models for the study of variant-specific tropism than conventional cell lines.

The selection of models provides a very good basis for the addressed studies and the manuscript covers a very broad spectrum of virus variants. Nevertheless, mechanistic analyses that could explain the postulated shift of variants from pulmonary to intestinal tropism are lacking. Thus, the work remains essentially descriptive. In addition, there are several problems regarding the chosen methods and the conduct of the experiments.

Major points:
Unfortunately, no accurate tropism analyses were performed in human lung/bronchial tissue. The microscopic evaluation should perform higher magnifications using the usual markers in immunofluorescence for the expected infected cell types (e.g. ATI/EMP2, ATII/HTII-280, AM/CD68), as well as in lung organoids. For tropism analysis in human lung tissue / bronchi and validation of lung organoids, it is also useful to show other infected cell types. For example, basal cells (p63/KRT5), secretory cells (MUC5AC) or ciliated cells (acTub/CAPS). It would also be very helpful if a semi-quantitative evaluation could be made about the number of SARS-CoV-2-positive cells in these models for each virus strain. Supplementary analyses, such as scRNA sequencing and the detection of subgenomic RNA to identify productive infection, would complement these investigations.
To conclude an actual shift in tropism and infection burden between the organ models, normalization of the replication data of the relevant variants in all models would have to be carried out. At best, several model types would be infected with the corresponding variants simultaneously. The choice of media, number of cells per model, moi etc. would also have to be very strictly controlled to validate the postulated pulmonary/intestinal shift.
The inhibitor experiments were only carried out with Vero-E6 cells and lung organoids. Since Caco-2 cells express significantly less ACE2 compared to intestinal enteroids, but comparable TMPRSS2, it would make sense to perform the inhibitor experiments on these cells. This comparison would support their hypothesis that BA.2.86 has a TMPRSS2-related advantage. Further, more application-related experiments, in which certain host factors are switched off or overexpressed to validate the results, are not included but are necessary (see above).
Immune responses were only measured in Caco-2 cells and intestinal enteroids. Human lung cells and lung organoids should also be included for reference and comparison especially as immune cells become involved, thus making the data more physiologically relevant.
The mRNA expression of TMPRSS2 and ACE2 in Figure S1 was measured, but to strengthen their hypothesis, it is crucial to assess actual protein expression (via Western blot and/or IHC). Additionally, this should be validated and compared across the most relevant models. Colocalization analyses of SARS-CoV-2-infected cells, ACE2 and TMPRSS2 would be

valuable.
The statistical analyses do not appear reliable. Many p-values have exactly the same value and significances have been measured, even if data points of two groups obviously do not or hardly differ from each other. A statistical assessment is necessary since many conclusions of the authors depend on that.

A mechanistic link explaining the shift of variants BA.2.86 and JN.1 from pulmonary to intestinal tropism would be very helpful to corroborate the descriptive data.

Minor issues:
The colors in the replication figures (Fig. 1 and 2) are difficult to distinguish. It would be advisable to adopt the presentation style of Fig. 4 and 5 (bar charts with individual data points). Care should be taken to ensure a consistent presentation of all subtypes in all figures and alignment of the figures. To keep things clear, it might be helpful to leave only the virus strains that are important for understanding the manuscript in the main figures, while all other data for the remaining strains are presented in the supplementary figures.
Input values are not listed. These should be measured, if necessary, and included in the figures.
The staining using a single antibody against the viral NP protein should be validated using additional antibodies against SARS-CoV-2 to ensure that different viral strains are covered equally by the antibodies.
Genes listed in qPCR part should be written in italics.
In Fig. 3, the spacing between individual images should be adjusted for consistency.
In Fig. 4, there are varying font sizes that need to be standardized.
Consider adding other variants with inhibitors to the Supplementary Information for Fig. 4.
In Fig. 5, the colors of the individual subtypes do not match within the same figure. Please refer to the previous point about color consistency.
Fig. 6D: The authors mention that only JN.1 does not show colocalization in goblet cells. Is this correct? Please clarify.
RRID numbers for the antibodies used should be included.
Line 68: Italicize "ex vivo" and "in vivo" here and throughout the manuscript.
Line 75: Should it be "more efficiently"?
Line 76: Should it be "publicationS" (plural)?
Line 82: Should it be "quantifying"?
Line 127: Usage of "Camostat mesylate" should be standardized throughout the manuscript.
Line 181: Should it be "other reportS" (plural)?
Line 191: Should it be "using" or "done in" for better clarity?
Line 205: The sentence is unclear; please revise for clarity.
Line 211: The sentence is unclear; please revise for clarity.
Lines 214/252: It's recommended to use "lung explant" or "lung tissue" consistently throughout the manuscript.
Line 217: The sentence is unclear; please revise for clarity.
Line 248: The sentence is unclear; please revise for clarity.
Line 272: Should all variants be capable of causing severe disease? Please clarify.

Reviewer #2

(Remarks to the Author)
I co-reviewed this manuscript with one of the reviewers who provided the listed reports. This is part of the Nature Communications initiative to facilitate training in peer review and to provide appropriate recognition for Early Career Researchers who co-review manuscripts.

Reviewer #3

(Remarks to the Author)
The manuscript by Hui et al. evaluated the replication ability of SARS-Cov-2 Omicron subvariants from BA.1 to JN.1 in cell lines, human airway (bronchus and lung) organoids, and intestinal (duodenum) enteroids. They found that EG.5.1, along with the more ancestral BA.2 strains replicated better than BA.2.86 and JN.1 in human airway organoids. However, BA.2.86 and JN.1 can replicate to high titers in intestinal enteroids compared to the EG.5.1, possibly due to the difference in ACE2 binding affinity. Overall, this is a comprehensive study on an important topic, providing a valuable asset to the very changing SARS-CoV-2 field. However, more experiments are needed to strengthen the conclusions and provide mechanistic depth.

Major comments:
1. The authors thoroughly evaluated the replication of different Omicron strains in human bronchus and lung organoids (Figures 1-3). However, only EG.5.1, BA2.86, and JN.1 were measured in the duodenal enteroids. It is difficult to determine, just based on this, whether this is a common feature shared by other subvariants. In addition, the authors critically need to test the replication kinetics of these viruses in colonoids as previously published (Jang KK et al., PLOS Biol, 2022; Antia A et al., Viruses, 2024, which should be referenced). This is especially important as intestine was used in the title of this manuscript to prove the observation is not intestinal segment-specific.
2. The latest Omicron subvariants harbor multiple mutations beyond the spike protein. To investigate whether the difference observed in airway vs intestinal enteroids is mediated by spike protein (ACE2 binding affinity dependent), the authors need to test the infectivity of different Omicron spikes in the context of either pseudotyped lentiviral vectors or recombinant vesicular stomatitis virus backbones as previously published. For simplicity, one representative subvariant from each group (for example EG.5.1 vs JN.1) is sufficient.

Minor concerns:
1. A diagram charting the evolutionary path of the Omicron lineage for all the strains tested in this study should be provided as part of Figure 1 for the readers.
2. Cellular marker co-staining with viral nucleoprotein in ex vivo cultures of human respiratory tissues should be provided in Figure 3 to show the cellular tropism as the subtitle suggested. Alternatively, tone down the statement and avoid the use of word tropism, which calls for the identification of specific cell types.
3. In figure 3a and 3b, the authors mentioned that more cells stained with viral nucleocapsid protein were observed in Omicron BA subvariants, XBB.1.5, BQ.1.22 and EG.5.1 than WT, BA.2.86 and JN.1 in human bronchial tissues while more positive cells were observed in BA.5, XBB.1.5, EG.5.1 and XBB.1.16 infection than the other variants in human lung tissues (Figure 3). Please make the results more quantitative or quantify the number of cells infected for each variant.
4. The virus input (1 hpi) was missing in Figure 4a and 4c.
5. Figure 6D, Villin is expressed in all intestinal epithelial cells and is not a specific marker for enterocytes, which should be replaced by SI, FABP1, or other brush border enzymes. Paneth cells and goblets cells staining look artificial to me. Please try to stain these three types of cells on the same sample using different pseudo colors.

Reviewer #4

(Remarks to the Author)
I co-reviewed this manuscript with one of the reviewers who provided the listed reports. This is part of the Nature Communications initiative to facilitate training in peer review and to provide appropriate recognition for Early Career Researchers who co-review manuscripts.

Version 1:

Reviewer comments:

Reviewer #1

(Remarks to the Author)
I would like to thank the authors for addressing the points raised. Although not all of the additional experiments could be carried out, I am satisfied with the reasoning presented and the current state of the manuscript.

Reviewer #2

(Remarks to the Author)
I co-reviewed this manuscript with one of the reviewers who provided the listed reports. This is part of the Nature Communications initiative to facilitate training in peer review and to provide appropriate recognition for Early Career Researchers who co-review manuscripts.

Reviewer #3

(Remarks to the Author)
The authors have satisfactorily addressed all the previous comments. No concerns.

Reviewer #4

(Remarks to the Author)
I co-reviewed this manuscript with one of the reviewers who provided the listed reports. This is part of the Nature Communications initiative to facilitate training in peer review and to provide appropriate recognition for Early Career Researchers who co-review manuscripts.

Open Access This Peer Review File is licensed under a Creative Commons Attribution 4.0 International License, which permits use, sharing, adaptation, distribution and reproduction in any medium or format, as long as you give appropriate credit to the original author(s) and the source, provide a link to the Creative Commons license, and indicate if changes were made.
In cases where reviewers are anonymous, credit should be given to 'Anonymous Referee' and the source.
The images or other third party material in this Peer Review File are included in the article's Creative Commons license, unless indicated otherwise in a credit line to the material. If material is not included in the article's Creative Commons license and your intended use is not permitted by statutory regulation or exceeds the permitted use, you will need to obtain permission directly from the copyright holder.
To view a copy of this license, visit https://creativecommons.org/licenses/by/4.0/

Response to Reviewers' comments
on Nature Communications manuscript NCOMMS-24-52853-T

<u>Reviewer #1: Comments and Suggestions for Authors</u>

The selection of models provides a very good basis for the addressed studies and the manuscript covers a very broad spectrum of virus variants. Nevertheless, mechanistic analyses that could explain the postulated shift of variants from pulmonary to intestinal tropism are lacking. Thus, the work remains essentially descriptive. In addition, there are several problems regarding the chosen methods and the conduct of the experiments.

Major points:

1: No accurate tropism analyses were performed in human lung/bronchial tissue. The microscopic evaluation should perform higher magnifications using the usual markers in immunofluorescence for the expected infected cell types (e.g. ATI/EMP2, ATII/HTII-280, AM/CD68), as well as in lung organoids. For tropism analysis in human lung tissue / bronchi and validation of lung organoids, it is also useful to show other infected cell types. For example, basal cells (p63/KRT5), secretory cells (MUC5AC) or ciliated cells (acTub/CAPS). It would also be very helpful if a semi-quantitative evaluation could be made about the number of SARS-CoV-2-positive cells in these models for each virus strain.

A: We thank the reviewer for this suggestion. From the panel of SARS-CoV-2 viruses, we chose the representative strains of each period for further detail analysis. We now include the co-staining of SARS-CoV-2 viral antigen together with cell markers for both human bronchial and lung tissue to address the cellular tropism of the representative variants in Figure 5. On the other hand, since immunohistology analysis is not a platform for comparing replication among different strain of virus, it is used mainly to show the difference in cellular tropism among different viruses. The gold standard for assessing viral replication still remains the $TCID_{50}$ *assay.*

2: Supplementary analyses, such as scRNA sequencing and the detection of subgenomic RNA to identify productive infection, would complement these investigations.

A: Thanks for the suggestion. However, we haven't established the scRNA analysis for tropism for ex vivo tissues. However, we agree that scRNA sequencing will be useful and in the future, we will establish this approach for future studies. Now we provide the cellular tropism by using immunofluorescence staining of viral antigen and cell markers as mentioned above.

3: To conclude an actual shift in tropism and infection burden between the organ models, normalization of the replication data of the relevant variants in all models would have to be carried out. At best, several model types would be infected with the corresponding variants simultaneously. The choice of media, number of cells per model, moi etc. would also have to be very strictly controlled to validate the postulated pulmonary/intestinal shift.

A: We agree that the infection condition of the tested viruses should be the same for a fair comparison. For the ex vivo infection, we used the same medium for incubation, same amount of virus input was used to infect both bronchial and lung explants. Proximal intestinal enteroids with 3D structures were incubated with specific medium as mentioned in

the Method section and we used the same amount of virus input for infection in the fixed amount of enteroids. These methods have been optimized and well established in our laboratory. The choice of media is specific to the model that it maintains the viability and the quality of cells; therefore, it might not be possible to use the same media for all the different models we used.

We carried out additional experiments using the 6 selected variants and infections were performed simultaneously for direct comparison in ex vivo bronchial and lung tissues, colon cells and proximal intestinal enteroids, and the results were presented in Figure 4, 6 and 7.

Although direct comparison of viral replication across different models might not be appropriate, we believe that comparison of the replication kinetics of different variants within the same model is valid. We found that BA.2.86 and JN.1 replicated to lower titres than EG.5.1 in both bronchial, and lung tissues, but in the GI tract, their replication were comparable in colon cells (BA.2.86 and JN.1 have 90% and 84% of EG.5.1 AUC, respectively) while in proximal intestinal enteroids BA.2.86 and JN.1 replicated to higher titres than EG.5.1 with 37% AUC compared to that of JN.1 (Figure 4a-d, 6a, 6b, 7a and 7b).

4: The inhibitor experiments were only carried out with Vero-E6 cells and lung organoids. Since Caco-2 cells express significantly less ACE2 compared to intestinal enteroids, but comparable TMPRSS2, it would make sense to perform the inhibitor experiments on these cells. This comparison would support their hypothesis that BA.2.86 has a TMPRSS2-related advantage.

A: We have performed additional inhibitor experiments in both colon cells and proximal intestinal enteroids using TMPRSS2 inhibitor (Camostat mesylate) and ACE2 inhibitor (Ouabain) with EG.5.1, BA.2.86 and JN.1 (Figure 9). We found that in colon cells, JN.1 was the most sensitive to both inhibitors compared to EG.5.1 and BA.2.86 while reduction of replication of BA.2.86 was slightly higher than EG.5.1 in the presence of the inhibitors. In proximal intestinal enteroids with higher ACE2 receptor expression than colon cells (Figure S2), TMPRSS2 inhibitor had similar effect of reduction in viral replication of BA.2.86 and JN.1 while ACE2 inhibitor reduced the replication of JN.1 to 9% compared to 38% of BA.2.86 (Figure 9b). These findings suggest that JN.1 replication in proximal intestinal enteroids is more dependent on ACE2 than TMPRSS2 and JN.1 is more dependent on ACE2 than BA.2.86. These results also suggest that JN.1 mainly uses ACE2 to enter cells in proximal intestinal enteroids while it may use other pathways for entry in colon cells.

6: More application-related experiments, in which certain host factors are switched off or overexpressed to validate the results, are not included.

A: We thank the reviewer for this suggestion. In order to reveal the involvement of ACE2 and TMPRSS2, we applied different inhibitors in the enteroids and colon cells as mentioned above.

5: Immune responses were only measured in Caco-2 cells and intestinal enteroids. Human lung cells and lung organoids should also be included for reference and comparison especially as immune cells become involved, thus making the data more physiologically relevant.

A: The reason for measuring immune response in colon cells and proximal intestinal enteroids is to investigate whether there are differences in cytokines and chemokines induction in the intestine between BA.2.86 and JN.1. Lower levels of cytokine and chemokine induction by JN.1 compared to that by BA.2.86 suggest that GI symptoms caused by JN.1 are probably less severe than BA.2.86 and this aids the spread of the virus through faecal route since infected person usually has not received any medical treatment. We have now included the

immune responses of the 6 selected virus strains in colon cells and proximal intestinal enteroids (Figure 6d and 7d).

6: The mRNA expression of TMPRSS2 and ACE2 in Figure S1 was measured, but to strengthen their hypothesis, it is crucial to assess actual protein expression (via Western blot and/or IHC).

A: We have performed the immunofluorescent staining of both TMPRSS2 and ACE2 in colon cells and proximal intestinal enteroids (Figure S1b and S1c) and we also measured the mRNA expression and immunofluorescent staining of ACE2 and TMPRSS2 in airway organoid, bronchial and lung tissues shown in the same figure (Figure S1).

7: Colocalization analyses of SARS-CoV-2-infected cells, ACE2 and TMPRSS2 would be valuable.

A: We have performed TMPRSS2 and ACE2 inhibitor experiments and showed that the viruses have different dependence on TMPRSS2 and ACE2 (Figure 9) as mentioned above in the response of question 4.

8: The statistical analyses do not appear reliable. Many p-values have exactly the same value and significances have been measured, even if data points of two groups obviously do not or hardly differ from each other. A statistical assessment is necessary since many conclusions of the authors depend on that.

A: We have double checked all the statistical analysis. The name of the test and post-test were listed, and the exact P values are indicated. They are performed using formulae in the Prism software.

9: A mechanistic link explaining the shift of variants BA.2.86 and JN.1 from pulmonary to intestinal tropism would be very helpful to corroborate the descriptive data.

A: In order to address this, we have performed additional experiments with different inhibitors. Based on our findings in colon cells and proximal intestinal enteroids including the viral replication kinetics, ACE2 dependency, and induction of immune response as mentioned above. A higher replication competence in small intestine of JN.1 and BA.2.86 than EG.5.1 probably contributes to the rapid expansion of these two variants over EG.5.1. While JN.1 induced less cytokines and chemokines than BA.2.86, it is more easily transmitted through faecal route as discussed above.

*As we provide evidence showing BA.2.86 and JN.1 having segment-specific replication advantages in the proximal intestine among the tested variants, we have revised the title of the manuscript to "**SARS-CoV-2 Omicron BA.2.86 and JN.1 expand tropism in human proximal intestinal epithelium**".*

More detailed findings and explanations are presented as follows:

"Based on our findings in colon cells, comparable levels of AUC were observed among the five virus strains, which had AUC lower than that of the WT, although statistical significance has been obtained between EG.5.1 and BA.2.86/JN.1 (Figure 6a and 6b). However, BA.1, BA.2.86 and JN.1 replicated to comparable levels as WT in proximal intestinal enteroids while XBB.1.5 and EG.5.1 had a limited replication competence than the other tested variants (Figure 7a and 7b). These findings suggest that variants have different replication competence in small proximal intestine and colon epithelial cells. In fact, many enteric viruses such as rotavirus and astrovirus, infect and replicate primarily in the small proximal

*intesti*ne causing viral shedding in stool leading to faecal-oral transmission[18,19] which implying the importance of tropism in small proximal intestine for faecal shedding and transmission. While clinical evidence shows that BA.2.86 and JN.1 infected patients have higher viral shedding in faecal samples when compared with patients infected with EG.5.1 and XBB.1.5[17], our findings suggest that the tropism and replication competence of SARS-CoV-2 in proximal intestinal enteroids is more essential than in colon cells for virus spreading through the faecal samples."*

Minor issues:

1: The colors in the replication figures (Fig. 1 and 2) are difficult to distinguish. It would be advisable to adopt the presentation style of Fig. 4 and 5 (bar charts with individual data points). Care should be taken to ensure a consistent presentation of all subtypes in all figures and alignment of the figures. To keep things clear, it might be helpful to leave only the virus strains that are important for understanding the manuscript in the main figures, while all other data for the remaining strains are presented in the supplementary figures. Input values are not listed. These should be measured, if necessary, and included in the figures.

A: The replication figures have been revised accordingly. The input values for each virus strain are the same which is 5 x 10^4 plaque forming units per tissue and this is now listed in the Method section.

2: The staining using a single antibody against the viral NP protein should be validated using additional antibodies against SARS-CoV-2 to ensure that different viral strains are covered equally by the antibodies.

A: We used two different antibodies for SARS-CoV-2 viral protein staining. One targeting *viral nucleocapsid (N) protein and the other targeting spike S1 domain. Both antibodies have been validated by the manufacturer being able to detect a number of variants including the SARS-CoV-2 WT, Alpha (B.1.1.7), Beta (B.1.351), Gamma (P.1), Delta (B.1.617.2), and Omicron (B.1.1.529). Our IHC and IFA staining also demonstrates the cross-reactivity of these antibodies against all the tested SARS-CoV-2 variants. N protein of SARS-CoV-2 is highly conserved among different variants.*

3: Genes listed in qPCR part should be written in italics.
In Fig. 3, the spacing between individual images should be adjusted for consistency.
In Fig. 4, there are varying font sizes that need to be standardized.
Consider adding other variants with inhibitors to the Supplementary Information for Fig. 4.
In Fig. 5, the colors of the individual subtypes do not match within the same figure. Please refer to the previous point about color consistency.

A: The gene names in qPCR have been changed to italics. The spacing in Figure 3 has been adjusted and font sizes in Figure 4 (now Figure S1) have been adjusted. We have performed additional experiments to include selected variants with inhibitors in airway organoids (Figure 4). The colors of the individual subtypes have been revised for consistency in Figure 1, 2, 4, 6, 7 and 10.

4: Fig. 6D: The authors mention that only JN.1 does not show colocalization in goblet cells. Is this correct? Please clarify.

A: *We apologize about this mistake, and we have revised the sentence based on our immunostaining results showing in Figure 8. We found that except XBB.1.5 in which no viral protein was stained, all the other tested variants show colocalization of viral protein in goblet cells which are indicated in the inserts (Figure 9).*

5: RRID numbers for the antibodies used should be included.

A: *We have included the RRID numbers of the antibodies used in Table S3.*

6: Line 68: Italicize "ex vivo" and "in vivo" here and throughout the manuscript.

Line 75: Should it be "more efficiently"?

Line 76: Should it be "publicationS" (plural)?

Line 82: Should it be "quantifying"?

Line 127: Usage of "Camostat mesylate" should be standardized throughout the manuscript.

Line 181: Should it be "other reportS" (plural)?

Line 191: Should it be "using" or "done in" for better clarity?

Line 205: The sentence is unclear; please revise for clarity.

Line 211: The sentence is unclear; please revise for clarity.

Lines 214/252: It's recommended to use "lung explant" or "lung tissue" consistently throughout the manuscript.

Line 217: The sentence is unclear; please revise for clarity.

Line 248: The sentence is unclear; please revise for clarity.

Line 272: Should all variants be capable of causing severe disease? Please clarify.

A: *We thank the reviewer for picking these up and the above have been revised accordingly.*

Reviewer #3: Comments and Suggestions for Authors

Overall, this is a comprehensive study on an important topic, providing a valuable asset to the very changing SARS-CoV-2 field. However, more experiments are needed to strengthen the conclusions and provide mechanistic depth.

Major comments:

1: The authors thoroughly evaluated the replication of different Omicron strains in human bronchus and lung organoids (Figures 1-3). However, only EG.5.1, BA2.86, and JN.1 were measured in the duodenal enteroids. It is difficult to determine, just based on this, whether this is a common feature shared by other subvariants. In addition, the authors critically need to test the replication kinetics of these viruses in colonoids as previously published (Jang KK et al., PLOS Biol, 2022; Antia A et al., Viruses, 2024, which should be referenced). This is especially important as intestine was used in the title of this manuscript to prove the observation is not intestinal segment-specific.

A: *We thank the reviewer for this suggestion. We have performed additional experiments including 6 representative SARS-CoV-2 variants in proximal intestinal enteroids. However, since colonoid cultures is not available in our laboratory, we have then investigated the replication kinetics of these 6 variants in colon cells.*

Through direct comparison of the cellular markers, the intestinal enteroids were characterized with elevated expression of proximal intestinal cell markers including lysozyme (LYZ), villin 1 (VIL1) and GATA binding protein 4 (GATA4) while Caco-2 cells had upregulated GATA binding protein 6 (GATA 6), which is expressed in colorectal epithelium (Figure S3a and S3b). Therefore, intestinal enteroids and Caco-2 cells represent proximal small intestine (proximal intestinal enteroids) and distal large intestine (colon cells), respectively.

Our findings in colon cells are consistent with previous reports in colonoid cutlures that Omicron/BA.1 infected less effectively than wild-type strain in colon epithelial cells and induced limited expression of both type I and III interferons, which were comparable to wild-type virus and mock infections (Antia et al, Viruses 2024). However, we found that WT induced higher levels of TNF-alpha than BA.1.

In the presence of ACE2 inhibitor, BA.2.86 and JN.1 replication reduced to 69% and 53% in colon cells, respectively, suggesting that factors other than ACE2 contributing to the infection and replication in colon cells. These findings are consistent with the other report working with colonoid derived cells infected with wild-type and Omicron spike protein pseudovirus (Jang et al., Plos Biol 2022).

Based on our findings in colon cells, comparable levels of AUC were observed among the five virus strains, which had AUC lower than that of the WT, although statistical significance has been obtained between EG.5.1 and BA.2.86/JN.1 (Figure 6b). However, BA.1, BA.2.86 and JN.1 replicated to comparable levels as WT in proximal intestinal enteroids while XBB.1.5 and EG.5.1 had a limited replication competence than the other tested variants (Figure 7a and 7b). These findings suggest that variants have different replication competence in small intestine and colon epithelial cells. In fact, many enteric viruses such as rotavirus and astrovirus, infect and replicate primarily in the small intestine causing viral shedding in stool leading to faecal-oral transmission[18,19] *which implying the importance of tropism in small intestine for faecal shedding and transmission. While clinical evidence shows that BA.2.86 and JN.1 infected patients have higher viral shedding in faecal samples when compared with patients infected with EG.5.1 and XBB.1.5*[17]*, our findings suggest that the tropism and replication competence of SARS-CoV-2 in proximal intestinal enteroids is more essential than in colon cells for virus spreading through the faecal samples.*

*As we provide evidence showing BA.2.86 and JN.1 having segment-specific replication advantages in the proximal intestine among the tested variants, we revised the title of the manuscript to "**SARS-CoV-2 Omicron BA.2.86 and JN.1 expand tropism in human proximal intestinal epithelium**".*

2: The latest Omicron subvariants harbor multiple mutations beyond the spike protein. To investigate whether the difference observed in airway vs intestinal enteroids is mediated by spike protein (ACE2 binding affinity dependent), the authors need to test the infectivity of different Omicron spikes in the context of either pseudotyped lentiviral vectors or recombinant vesicular stomatitis virus backbones as previously published. For simplicity, one representative subvariant from each group (for example EG.5.1 vs JN.1) is sufficient.

A: *By using ouabain to inhibit the ACE2 binding to the spike protein of SARS-CoV-2 virus, we demonstrate the significant reduction in viral titre of BA.2.86 and JN.1 in proximal intestinal enteroids to 38% and 9%, respectively, while replication of BA.2.86 and JN.1 in colon cells*

was only partially reduced to 69% and 53%, respectively (Figure 9). These findings indicate that the two variants are more dependent on ACE2 binding in proximal intestinal enteroids than in colon cells.

Minor concerns:

1: A diagram charting the evolutionary path of the Omicron lineage for all the strains tested in this study should be provided as part of **Figure 1** for the readers.

A: A diagram showing the evolutionary path of the Omicron lineage has been added as Figure 1a. In addition, we summarize the tissue tropism of the viruses in Figure 10.

2: Cellular marker co-staining with viral nucleoprotein in ex vivo cultures of human respiratory tissues should be provided in **Figure 3** to show the cellular tropism as the subtitle suggested. Alternatively, tone down the statement and avoid the use of word tropism, which calls for the identification of specific cell types.

A: We have performed the co-staining of viral nucleocapsid protein in ex vivo bronchial and lung tissues to identify the cellular tropism of 6 representative virus strains in Figure 5.

3: In **figure 3a and 3b**, the authors mentioned that more cells stained with viral nucleocapsid protein were observed in Omicron BA subvariants, XBB.1.5, BQ.1.22 and EG.5.1 than WT, BA.2.86 and JN.1 in human bronchial tissues while more positive cells were observed in BA.5, XBB.1.5, EG.5.1 and XBB.1.16 infection than the other variants in human lung tissues (Figure 3). Please make the results more quantitative or quantify the number of cells infected for each variant.

A: We would like to use the immunofluorescence staining to confirm the presence of viral antigen and it can serve to provide cellular tropism based on the cell morphology of infected cells. $TCID_{50}$ assay serves as the gold standard to quantify the amount of virus replication in the bronchial and lung tissues. Regarding the cellular tropism, we now provide co-staining of viral antigen and cellular markers in the bronchial and lung tissues infected with six representative virus strains in Figure 5a and 5b.

4: The virus input (1 hpi) was missing in **Figure 4a and 4c**.

A: We thank the reviewer for picking this up. The viral titres at 1 hpi of original Figure 4a and 4c (now Figure S1a and S1c) were undetectable, therefore, we did not show this in the bar chart. We now added this information in the figure legend.

5: **Figure 6D,** Villin is expressed in all intestinal epithelial cells and is not a specific marker for enterocytes, which should be replaced by SI, FABP1, or other brush border enzymes. Paneth cells and goblets cells staining look artificial to me. Please try to stain these three types of cells on the same sample using different pseudo colors.

A: We thank the reviewer for this comment. We now stained FABP1 as the marker for intestinal epithelial cells together with the viral antigen as shown in Figure 8. Although the color or brightness of the immunofluorescence staining might look artificial, we believe that the presence or absence of the staining is accurate and the brightness/intensities will not be used as a quantification tool but they serve to confirm the co-localization of viral antigen and specific cell markers.
